# Sustainable MXene/Conductive Cellulose Heteroinks for 3D Printed High Areal Energy Density Micro‐Supercapacitors and Self‐Powered Integrated Systems

**DOI:** 10.1002/advs.202511439

**Published:** 2025-08-12

**Authors:** Chunling Cao, Shiyao Tang, Xiaofei Wu, Haibo Huang, Shouxin Liu, Hongpeng Li

**Affiliations:** ^1^ Key Laboratory of Bio‐Based Material Science and Technology (Ministry of Education) Northeast Forestry University Harbin 150040 China; ^2^ College of Mechanical Engineering Yangzhou University Yangzhou 225127 China; ^3^ School of Automotive Engineering Nantong Institute of Technology Nantong 226001 China

**Keywords:** 3D printing, conductive cellulose, MXene, micro‐supercapacitors, self‐powered integrated system

## Abstract

The additive manufacturing of micro‐supercapacitors (MSCs) with outstanding areal energy density and scalable integration remains challenging due to the incompatibility between printability and functionality of electronic ink. Here, a thixotropic MXene/conductive cellulose heteroink is formulated, eliminating the need for tedious processing and toxic organic additives, to construct MSCs with high areal energy density. Conductive cellulose with radially graded structure containing defect‐rich graphitic shells not only inhibits MXene re‐stacking through hydrogen‐bonded 3D porous networks, but also establishes sp^2^‐carbon pathways for rapid electron transport. The optimized 3D printed MSCs achieve record‐breaking metrics: high areal capacitance of 3.12 F cm^−2^ (1 mA cm^−2^), outstanding energy density of 1.25 mWh cm^−2^, and 95% capacitance retention after 10 000 bending cycles. Notably, the 3D printed MSCs can operate stably within a temperature range of −40 to 60 °C. In addition, an integrated flexible sensing system incorporating 3D printed MSCs and strain sensors is demonstrated, which is highly sensitive for real‐time motion monitoring. This work establishes a materials‐by‐design paradigm for customizable micro‐energy systems, advancing wearable and implantable electronics.

## Graphical abstract

A thixotropic MXene/conductive cellulose ink is developed for 3D printed micro‐supercapacitors (MSCs) that deliver ultrahigh areal capacitance, superior energy density, and exceptional flexibility. The hydrogen‐bonded 3D network effectively suppresses MXene restacking, while graphene‐like sp^2^carbon pathways accelerate electron transport, boosting electrochemical performance. The resulting MSC operates reliably under extreme conditions and enables self‐powered integrated wearable surveillance systems.

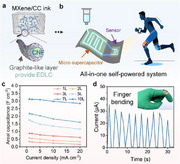

## 1 Introduction

The rapid proliferation of wearable and implantable electronics has intensified the demand for microscale energy storage systems that harmonize high energy density, mechanical resilience, and seamless integration.^[^ ^1^ ^]^ Micro‐supercapacitors (MSCs) have emerged as a focal point of interest owing to their noteworthy attributes, including high‐power density, rapid charge‐discharge capability, and prolonged cycle life.^[^ ^2^, ^3^ ^]^ However, their practical deployment is constrained by low areal energy density, limited scalability in electrode fabrication, and challenges in modular integration.^[^ ^4^ ^]^ 3D printing technology is greatly esteemed for its capacity to produce customizable microdevices with high areal energy densities.^[^ ^5^ ^]^ By meticulously engineering the micropatterns and precisely managing the printed thickness, the application of printable inks allows for highly accurate tuning of both the mass loading and geometry of microelectrodes.^[^ ^6^, ^7^ ^]^ Nevertheless, the development of multifunctional inks that simultaneously ensure high electrochemical performance, structural fidelity, and processability remains a pivotal challenge.^[^ ^8^, ^9^ ^]^

MXenes, particularly Ti_3_C_2_T*_x_*, have garnered significant attention as electrode materials due to their metallic conductivity, pseudocapacitive behavior, and tunable surface chemistry.^[^ ^4^, ^5^, ^6^, ^7^, ^8^, ^9^, ^10^ ^]^ These properties are crucial for the development of high‐precision 3D printing inks and the construction of MSCs with high energy density.^[^ ^11^, ^12^ ^]^ Nevertheless, their practical utility in 3D printed MSCs is hampered by MXene nanosheet restacking driven by van der Waals forces, which diminishes accessible active sites, impedes ion diffusion, and degrades ink rheology.^[^ ^13^ ^]^ Prior strategies, such as integrating surfactants or non‐conductive cellulose nanofibers (CNFs), partially alleviate restacking but compromise conductivity or fail to augment capacitance.^[^ ^14^ ^]^ For instance, by introducing the amphiphilic surfactant nonaethylene glycol monododecyl ether into the MXene gel, Shi et al. tailored the ink's rheology, significantly improving viscoelasticity and facilitating high‐resolution extrusion printing of precisely controlled architectures.^[^ ^15^ ^]^ Compared with pure MXene hydrogel printed MSC, it showed a high capacity of 1.58 F cm^−2^ at a scan rate of 5 mV s^−1^. Zhou et al. employed CNFs to modulate rheology but sacrificed conductivity, underscoring the persistent trade‐off between ink processability and electrochemical performance.^[^ ^16^ ^]^ These limitations highlight the urgent need for a multifunctional additive capable of concurrently addressing MXene restacking, enhancing conductivity, and optimizing ink rheology.

Herein, we present a breakthrough in MXene‐based ink design through the integration of conductive cellulose (CC). The CC architecture featuring a graphene‐mimetic outer sheath and cellulose core enables three synergistic functions: 1) suppression of MXene restacking via hydrogen‐bonded 3D porous networks, simultaneously enhancing intermolecular interactions, energy dissipation during deformation, and flexibility; 2) establishment of percolating sp^2^‐carbon pathways via the graphene‐like layer, facilitating rapid electron transport and significantly boosting electrochemical performance; and 3) imparting shear thinning rheology essential for high‐fidelity printing. This multifunctional design surmounts the longstanding trade‐off between ink processability and electrochemical efficacy in printed energy devices. This multifunctional architecture resolves the longstanding conflict between ink processability and electrochemical efficacy. The 3D printed MXene/CC (MC) MSCs deliver a record areal capacitance of 3.12 F cm^−2^ at 1 mA cm^−2^ and an energy density of 1.25 mWh cm^−2^, surpassing all reported MXene‐based printed MSCs. Furthermore, the 3D printed MSCs could work steadily across extreme temperatures (−40 to 60 °C), addressing the critical need for scalable, high‐voltage microsystems. By seamlessly coupling MC‐MSCs with pressure sensors, we demonstrate an all‐in‐one self‐powered integrated system capable of real‐time motion monitoring, underscoring its versatility in wearable applications. This work pioneers a materials‐by‐design paradigm that bridges additive manufacturing with high‐performance energy storage, paving the way for customizable, multifunctional microelectronics.

## 2 Results and Discussion

### 2.1 Fabrication of MC‐MSC and All‐In‐One Self‐Powered Sensing System

**Figure 1:**
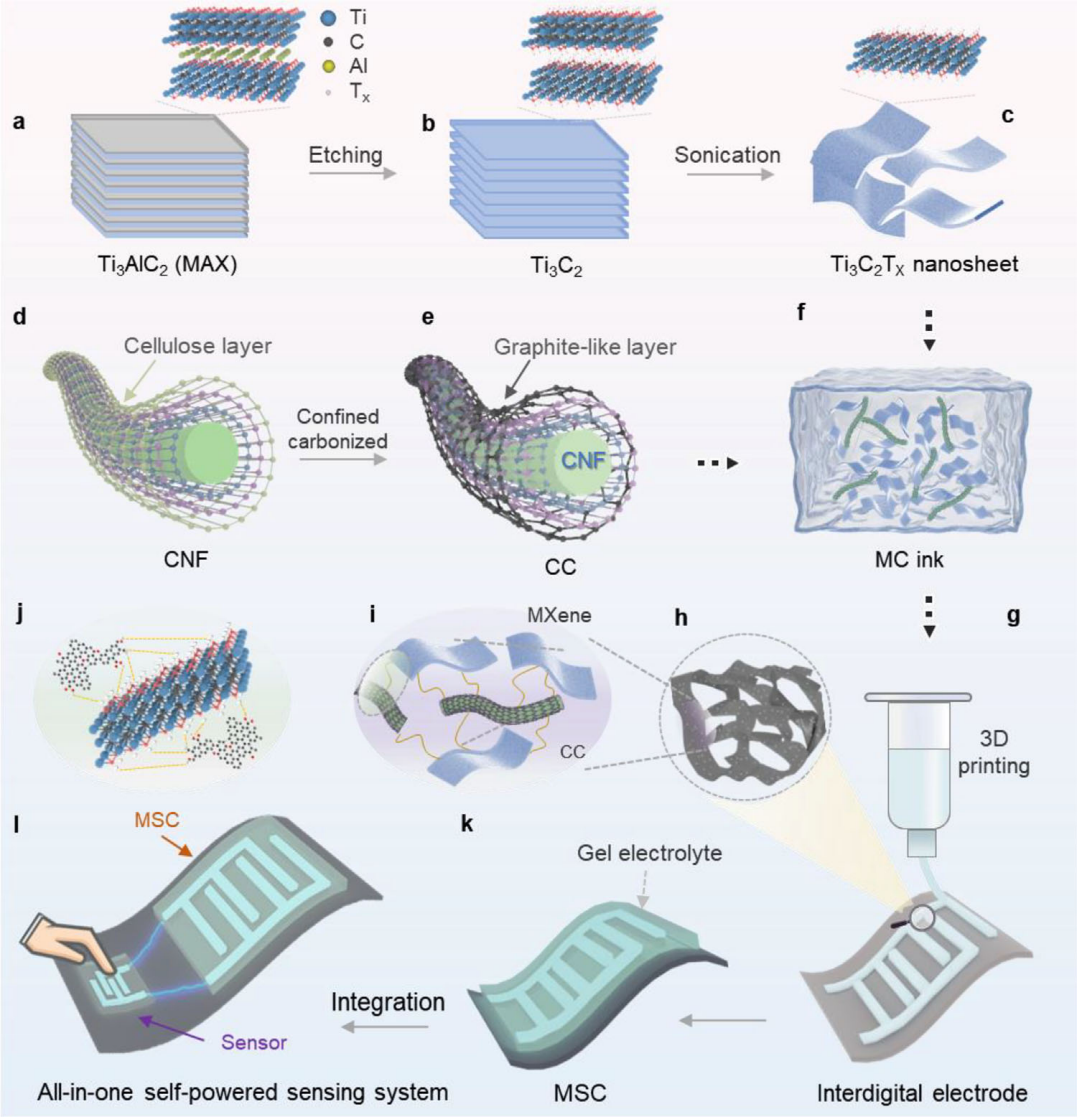
Design and fabrication of MC‐MSCs and a self‐powered integrated sensing system manufacturing. a–c) Synthesis of MXene nanosheets. d, e) Preparation of CC. f–k) 3D printed MC‐MSCs. l) All‐in‐one self‐powered system.

The fabrication and integration of MC‐MSCs with self‐powered sensing functionality were achieved through a rationally designed 3D printing strategy, as illustrated in **Figure** **1**. The process commenced with the preparation of the MC ink, achieved through the homogeneous dispersion of MXene nanosheets and CC in deionized water. This ink served as the cornerstone for the subsequent printing of MSC electrodes and sensors using the direct ink writing (DIW) 3D printed technique (Figure 1a–f). Subsequently, the resulting printable MC ink was transferred into a syringe and extruded through the needle under controlled pressure to print interdigitated in‐plane microelectrodes onto the substrate (Figure 1g). The aqueous MC ink demonstrates tunable viscosity optimized for substrate compatibility, allowing direct printing of high‐fidelity patterns on diverse substrates to fabricate MSCs with tailored geometries. CC is uniformly distributed across the MXene surface, and the interaction between CC and MXene, which is rich in surface oxygen‐containing functional groups, leads to the formation of a network structure through hydrogen bonding (Figure 1h–j). Notably, the presence of CC in the printed microelectrodes effectively mitigates the restacking of MXene layers and creates conductive tunnels that facilitate charge transfer. Upon drying of the microelectrode, a gel electrolyte was dropwise added to form the MC‐MSC (Figure 1k). In addition to the application of MSCs for energy storage, the MC ink is also harnessed for the design of high‐performance pressure sensors. Leveraging the exceptional precision of DIW printing, the MC ink is strategically deposited onto the same substrate as the MSCs, achieving seamless integration between the two components. This series of meticulously orchestrated steps not only yields MSCs that are both flexible and self‐powered but also seamlessly incorporates pressure sensing functionality, collectively constructing an integrated system with unified functionality.

### 2.2 Structural Characterization of MXene and CC

The synthesis of MXene nanosheets was achieved through selective etching of Ti_3_AlC_2_ MAX phase in LiF/HCl solution, followed by delamination in deionized water. X‐ray diffraction (XRD) analysis confirmed the successful removal of the MAX phase, evidenced by the disappearance of the original (002) peak at ≈9.5° (Figure S1, Supporting Information). The emergence of a new (002) reflection at 7.1° with reduced full‐width‐at‐half‐maximum (1.2°) confirms the formation of few‐layer MXene nanosheets, consistent with prior reports.^[^ ^17^ ^]^ Atomic force microscopy and transmission electron microscopy (TEM) characterization revealed MXene nanosheets with lateral dimensions of 250–400 nm and ultrathin thickness (≈2.5 nm), verifying successful exfoliation of Ti_3_AlC_2_ MAX phase into few layer MXene.

**Figure 2:**
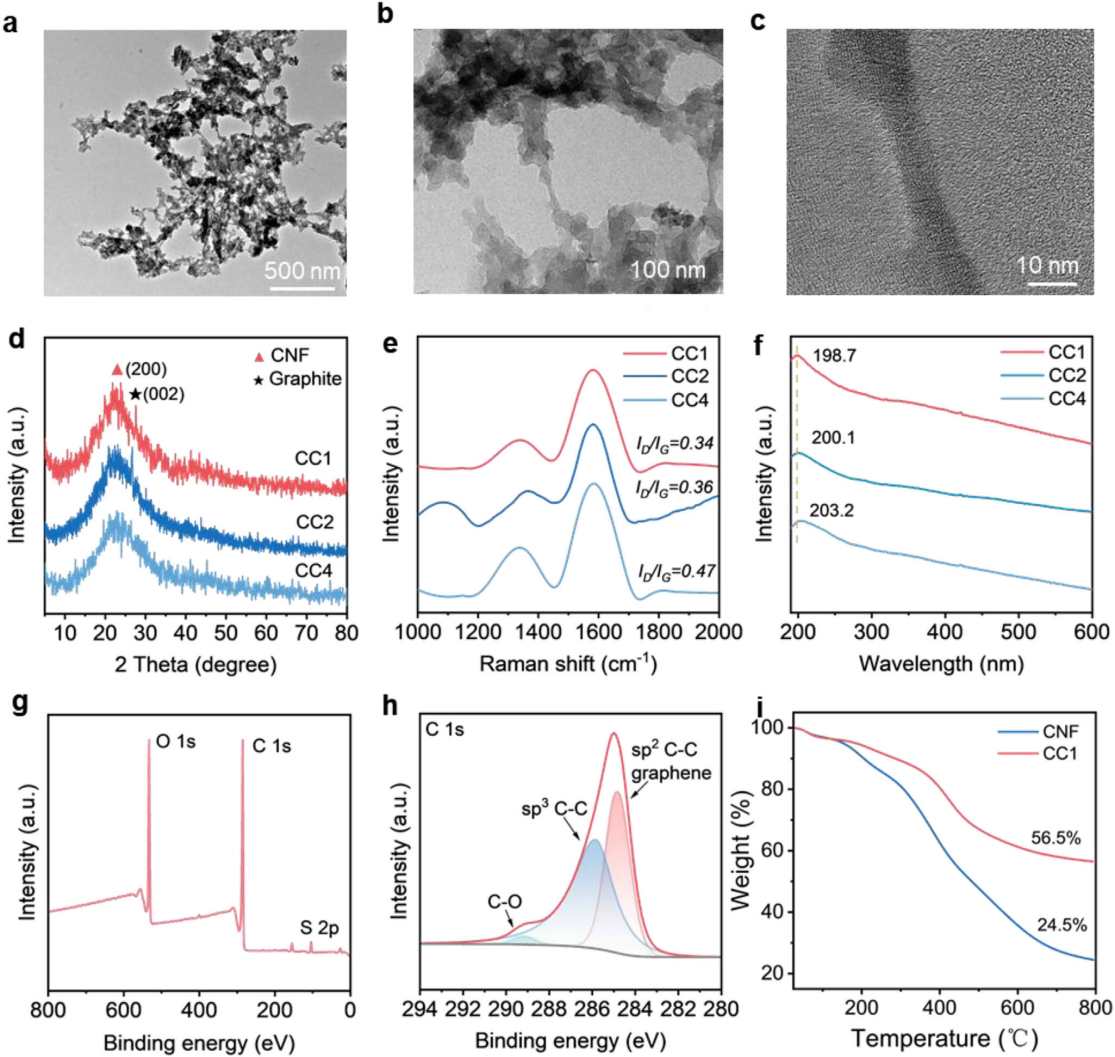
Characterizations of CC. a, b) TEM image, and c) HRTEM image of CC1. d) XRD patterns, e) Raman image, and f) UV–vis spectra of CC with different DC reaction times. g) XPS survey spectrum, and h) C 1s spectra of CC. i) TGA curve of CC.

To avoid the stack issue of MXene nanosheets caused by Van der Waals forces, as well as the subsequent reduction in slurry flowability and hindered ink extrusion, we introduced CC synthesized via an innovative DC method as an additive to fabricate MC‐MSCs. Unlike conventional graphite‐dependent approaches requiring high temperature treatments (>1000 °C), this one‐step DC process enables gram‐scale production of CC from bamboo‐derived CNF under ambient pressure using concentrated sulfuric acid.^[^ ^18^, ^19^ ^]^ TEM analysis (**Figure** **2**a‐c) reveals interconnected CC networks with distinct core‐shell structures, where graphitic carbon layers (d‐spacing = 0.34 nm) encapsulate CNF cores. The XRD pattern of CC1 (1 h DC treatment) reveals a dual‐phase crystalline structure (Figure 2d), characterized by a residual CNF (200) reflection at 21.8° and a graphitic carbon (002) peak at 27.1°.^[^ ^20^ ^]^ Prolonged DC treatment (CC2: 2 h; CC4: 4 h) progressively diminishes the graphitic (002) peak intensity, suggesting partial structural degradation under extended carbonization conditions.^[^ ^21^ ^]^ The CC displayed a quintessential graphitized structure, suggesting that the ideal condition for the formation of graphene layers is achieved through a 1 h DC process.

Raman spectroscopy further elucidates the structural evolution of CC (Figure 2e). The characteristic D‐band (1363 cm^−1^, disordered carbon) and G‐band (1581 cm^−1^, graphitic sp^2^ domains), respectively. The CC1 relative intensity ratio (I_D_/I_G_) was 0.34, indicating a high degree of graphitization and structural regularity.^[^ ^22^ ^]^ The presence of D‐bands was attributed to defective or disordered structures stemming from oxygen‐containing functional groups.^[^ ^23^ ^]^ UV‐vis spectra (Figure 2f) reveal broad absorption across 200–800 nm for CC, resembling graphene derivatives and multi‐walled carbon nanotubes, suggesting similar π‐π* electronic transitions.^[^ ^24^ ^]^ X‐ray photoelectron spectroscopy (XPS) confirms progressive decarbonization during DC treatment: the C─O content decreases from 25% (CNF) to 2% (CC1), while sp^2^‐hybridized C‐C content increases to 42% (CC1) (Figure 2g,h). Notably, residual sulfur originates from sulfonic acid groups introduced during the DC process, which may enhance interfacial interactions with MXene. Thermogravimetric analysis (TGA) demonstrates significantly improved thermal stability for CC versus pristine CNF (Figure 2i), with 56.5% mass retention at 800 °C versus 24.5% for CNF, attributable to protective graphitic shells. Combined structural analyses establish that the 1 h DC treatment optimizes graphitic layer formation while preserving structural integrity, which is critical for balancing conductivity and mechanical stability in composite inks.

### 2.3 Rheology and Printability of MC Ink

**Figure 3:**
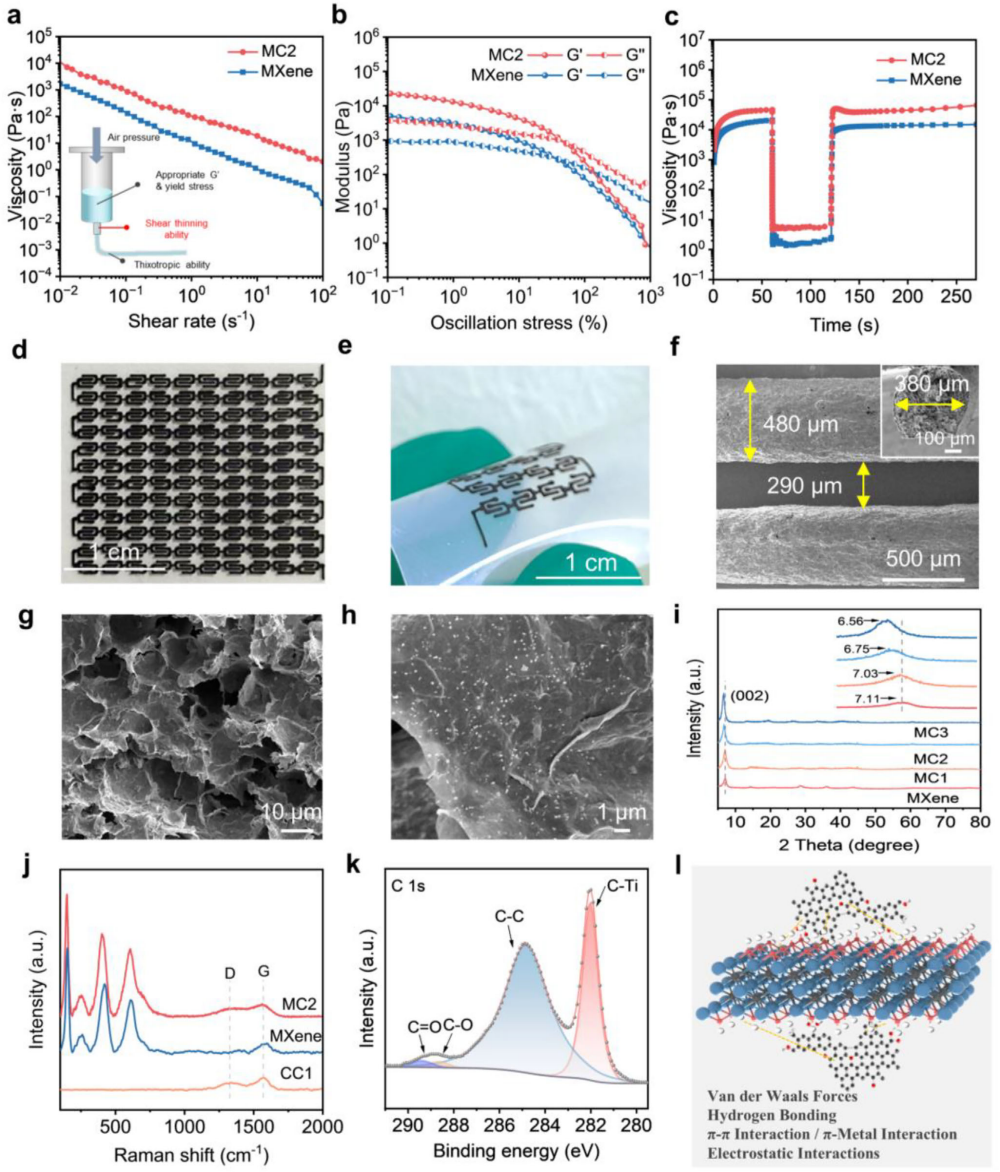
Rheology and printability of the MC ink. a) Shear rate dependent viscosity profiles, b) storage modulus (G′) and loss modulus (G″) as a function of oscillatory stress, and c) thixotropic recovery tests for MXene and MC2. d) Photograph of a large‐scale 120 cells integrated array and e) flexible monolithic MC‐MSC device. f‐h) SEM images of the interdigitated electrode. i) XRD patterns of MC (inset: The enlarged XRD pattern of the MXene (002) peak). j) Raman spectra of MC2, CC and MXene. k) High resolution C 1s XPS spectra deconvoluting chemical bonding states in MC2. l) Scheme of interaction between MXene nanosheet and CC.

Integration of MXene with CC yielded a viscoelastic 3D printable ink after controlled solvent evaporation. The rheological properties of the ink, critical for achieving high‐fidelity extrusion‐based printing, were systematically investigated (**Figure** **3**a–c). Both MXene and the MC2 inks exhibited pronounced shear‐thinning behavior (Figure 3a), a hallmark of thixotropic fluids essential for extrusion through fine nozzles (shear rate < 10^2^ s^−1^) and shape retention post‐deposition (shear rate > 10^−^^2^ s^−1^).^[^ ^25^ ^]^ Compared to pure MXene ink, the MC ink exhibits a rapid increase in solution viscosity due to the “crosslinking” effect between the added CC and MXene. Moreover, as the quantity of CC rose, the viscosity of the MC ink incrementally increased (Figure S2a, Supporting Information). Oscillatory stress sweeps demonstrated the superior mechanical stability of the MC2 ink, where the storage modulus (G′) consistently exceeded the loss modulus (G′′) below a yield stress of 959.4 Pa (Figure 3b), ensuring structural integrity during printing. Upon exceeding this yield stress, the ink transitioned from predominantly elastic (G′ > G′′) to viscous‐dominated behavior (G′′ > G′), enabling smooth extrusion under high shear stress while maintaining structural fidelity.^[^ ^26^ ^]^ Notably, both the plateau modulus and yield stress increased systematically with higher CC loading (Figure S2b, Supporting Information). The MC2 ink exhibited a maximum plateau G′ of 10989 Pa, which is optimal for achieving high printing resolution and fabricating stable, free‐standing architectures.^[^ ^27^ ^]^ To simulate the DIW process, we performed peak‐hold tests to characterize the shear‐rate‐dependent rheological behavior of the optimized MC2 ink (Figure 3c; Figure S2c, Supporting Information). When the shear rate undergoes a sudden increase, the viscosities of both MC and MXene inks undergo an abrupt reduction. Subsequently, as the shear rate returns to 0.01 s^−1^, the viscosities swiftly revert to their original levels, indicating the remarkable elasticity of their rheological properties. In addition, the effect of CC with different reaction times on rheological properties was evaluated. As can be seen from Figure S3 (Supporting Information), with the extension of the DC reaction time of CC, its regulatory effect on the rheology of the ink gradually weakens. This may be attributed to the excessive reaction time of CC, leading to a reduction in oxygen‐containing functional groups and a weakening of hydrogen bonding interactions, which in turn decreases its water retention capacity and subsequently reduces its viscoelasticity.^[^ ^28^ ^]^

In this context, 3D printed MSCs have several advantages, including large‐scale preparation (Figure 3d), and the ability to seamlessly integrate on the same substrate (Figure 3e). Various 2D planar patterns and 3D structures are successfully printed with high geometric accuracy (Figure S4, Supporting Information). A systematic investigation into the morphological characteristics and microstructural evolution of interdigital electrodes in MC‐MSCs was conducted through comprehensive microscopic analysis (Figure S5, Supporting Information). As evidenced by the SEM images in Figure 3f–h, the fabricated electrodes exhibit exceptional morphological uniformity with well defined linear architecture, demonstrating the remarkable thixotropic behavior and intrinsic self‐supporting capability of the MC ink formulation. To ensure structural uniformity, our printing process employs precise parameter optimization: ink concentration, nozzle diameter (410 µm), pressure (50 kPa), and speed (1 mm s^−1^) collectively enable consistent fabrication of 480 µm diameter electrodes, where the 17% diameter increase versus nozzle size stems from controlled post extrusion swelling. The optimized geometrical parameters establish an efficient conductive network that significantly promotes rapid ion/electron transport kinetics. Cross sectional analysis reveals a quasi‐semicircular electrode profile with densely distributed macropores (10–20 µm diameter) throughout the structure (Figure 3f). This distinctive hierarchical porosity in MC2 electrodes, as confirmed by SEM characterization, originates from the controlled ice templating process where stochastic nucleation and subsequent directional sublimation of ice crystals within the ink matrix create interconnected pore channels.^[^ ^29^, ^30^ ^]^ Furthermore, it can be observed in MC2 inks that CC is uniformly distributed above MXene (Figure 3g,h; Figure S6, Supporting Information). However, increased CC content beyond the critical threshold induces agglomeration phenomena due to competing electrostatic interactions between constituent materials (Figure S7, Supporting Information). Comparative analysis with pure MXene electrodes (Figure S8, Supporting Information) highlights the structural superiority of MC2 composites, where CC components serve as effective interlayer spacers to mitigate MXene nanosheet restacking while simultaneously enhancing electrolyte accessibility through the engineered porous architecture.

The chemical structure and molecular interactions of the composites were further studied by XRD, as shown in Figure 3i. All samples showed characteristic peaks of MXene. As CC content is added, the characteristic peak of MXene (002) appears to be shifted to a lower angle, indicating an increase in the interlayer spacing of the MXene nanosheets, demonstrating the successful insertion of CC into the interlayer of the MXene nanosheets.^[^ ^10^ ^]^ The Raman spectrum of MXene exhibits characteristic vibrational modes at 151 and 715 cm^−1^ (Figure 3j), corresponding to out‐of‐plane vibrations of Ti and C atoms, respectively. Additionally, the peaks observed at 261, 422, and 610 cm^−1^ are assigned to in‐plane vibrational modes involving Ti atoms, C atoms, and surface functional groups (e.g., ─O, ─F, or ─OH),^[^ ^31^ ^]^ respectively. The peaks at 1385 and 1569 cm^−1^ for the MXene sample are characteristic of the D‐band and G‐band of graphitic carbon, which are attributed to exposed C on the surface of MXene.^[^ ^32^ ^]^ In the Raman spectrum of MC2, the D‐band, and G‐band can be identified, which demonstrates that CC was successfully intercalated between MXene. Moreover, other characteristic peaks in MC2 showed a left shift compared with pure MXene, indicating a strong interaction between MXene and CC.^[^ ^29^ ^]^

X‐ray photoelectron spectroscopy (XPS) further elucidated chemical bonding evolution (Figure 3k; Figures S9, and S10, Supporting Information). The MC2 survey spectrum confirmed elemental coexistence of C (284.8 eV), Ti (454.3 eV), O (530.3 eV), and F (683.9 eV). High‐resolution C 1s deconvolution revealed enhanced oxygen functionality in MC2, with C─O and C ═ O bond energies upshifted to 288.64 and 289.40 eV, respectively, versus pristine MXene (288.30, 289.09 eV).^[^ ^33^ ^]^ Concurrently, O 1s spectra exhibited a 0.22 eV positive shift (531.44 eV for MC2 vs 531.22 eV for MXene), corroborating that strengthened oxygen‐mediated interfacial connectivity is well connected with CC. Ti 2p orbital analysis identified chemical states (Ti‐C: 455.03 eV; Ti─O: 458.89 eV; Ti‐F: 460.68 eV), verifying preserved surface terminations that enable hydrogen bonding interactions with CC. Collectively, these spectroscopic findings establish a multiscale interfacial engineering mechanism where CC incorporation modulates MXene interlayer spacing while fostering covalent and non‐covalent interactions through oxygen bridges and hydrogen bonding networks (Figure 3l).

### 2.4 Electrochemical Performance of MC‐MSCs

**Figure 4:**
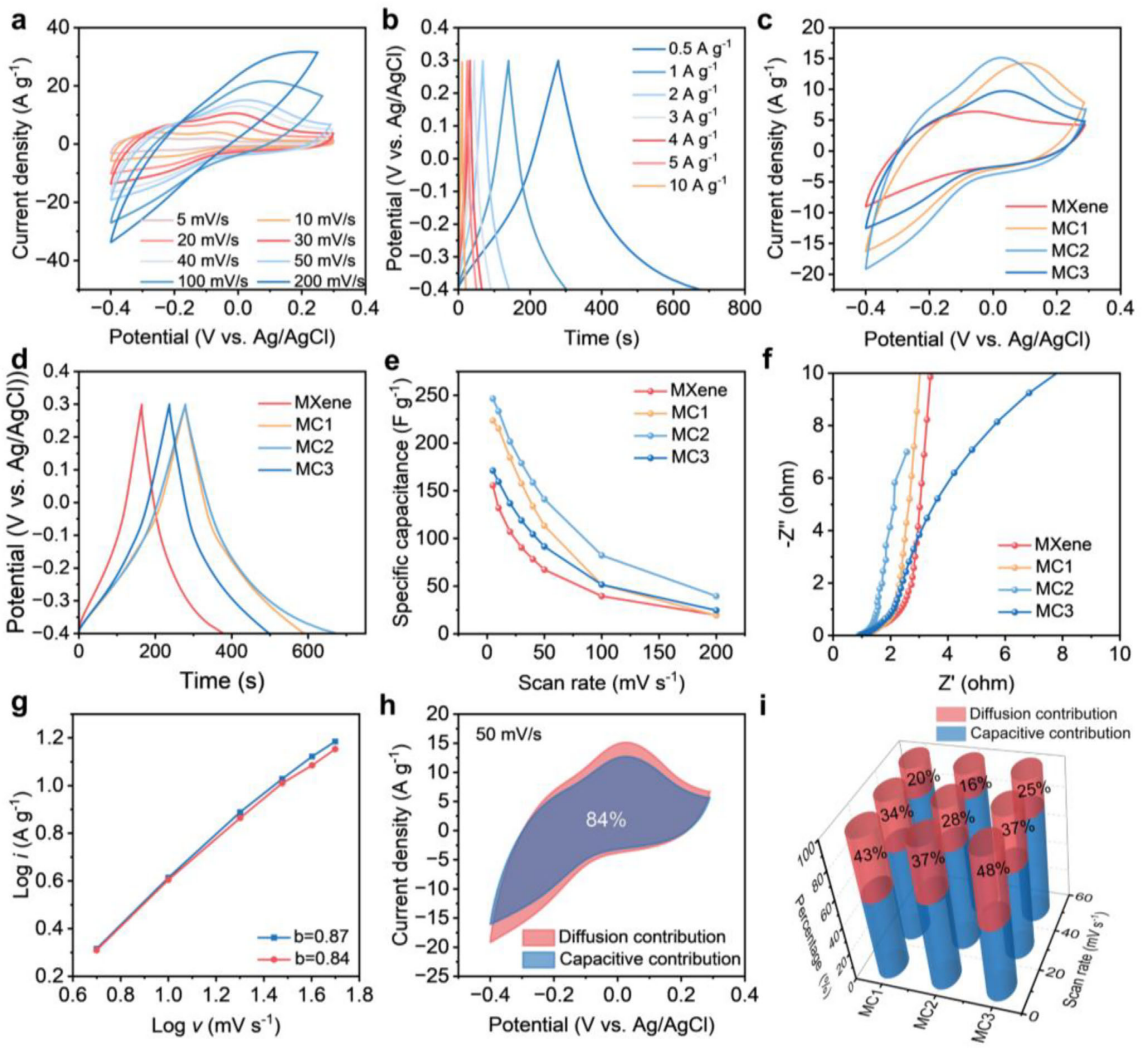
The electrochemical performance of the film electrodes obtained by the three‐electrode system. a) CV profiles, and b) GCD profiles of MC2. c) CV profiles at 50 mV s^−1^, d) GCD profile at 0.5 A g^−1^, e) specific capacitance at various scan rates, and f) Nyquist plots of MXene and MC electrodes. g) Logarithm plot of peak currents as a function of scan rates, h) Capacitive contribution to charge storage (50 mV s^−1^), and i) percentage of capacitance contribution and diffusion controlled for MC‐2 electrode.

The electrochemical characteristics of MC and MXene electrodes were systematically investigated in 1 M H_2_SO_4_ electrolyte using a three electrode configuration. Cyclic voltammetry (CV) revealed distinct quasi‐rectangular profiles for MC electrodes (**Figure** **4**a), suggesting capacitive behavior at a pseudoconstant rate over the complete voltammetric cycle. Galvanostatic charge‐discharge (GCD) measurements demonstrated near‐symmetrical triangular curves across current densities of 0.5 to 10 A g^−1^ (Figure 4b), confirming the exceptional reversibility and pseudocapacitive contributions inherent to MXene‐based systems. Notably, the expanded CV curve integration area of MC electrodes versus MXene counterparts (Figure 4c,d) quantitatively verified enhanced specific capacitance, with MC2 achieving 246.5 F g^−1^ at 5 mV s^−1^ compared to 155.5 F g^−1^ for pure MXene (Figure 4e). The enhanced electrochemical performance of the MC electrode relative to pure MXene is primarily attributed to the incorporation of highly conductive carbon chains within the ink, which establishes efficient electron transport pathways while maintaining structural integrity. The graphene‐like structure of the outer layer of CC provides effective electric double‐layer capacitance (EDLC), while the charge storage mechanism of MC electrodes is a combination of faradaic and EDLC storage. In addition, the contribution of CC4 to the capacity of MCC4 (CC4: MXene = 2:8) is not large, mainly because the carbon content of CC4 is relatively small (Figure S11, Supporting Information). In the MC composite, the CC component serves as a nanoscale spacer that simultaneously addresses two critical challenges: 1) exposing additional electrochemically active surfaces by mitigating MXene nanosheet restacking, while 2) establishing continuous, highly conductive networks that facilitate rapid electron transport and ion diffusion. This is further demonstrated by the EIS spectra and electrical conductivities of the MC and MXene electrodes (Figure 4f). From the Nyquist plots, it is clear that the charge in the MC2 electrode migrates at high frequencies and has lower ion diffusion resistance at low frequencies.

The charge storage kinetics were quantitatively analyzed using the power‐law relationship (Equation 1):^[^ ^34^, ^35^ ^]^

(1) $$i=av^{b}$$

where *i* represents the peak current, *v* is the scan rate, and *a* is a constant. The exponent b was determined from the slope of log(*i*) versus log(*v*) plots. A *b‐value* of 0.5 indicates diffusion‐controlled processes, while *b = 1.0* corresponds to ideal capacitive behavior. The *b*‐value corresponding to the two peak currents is calculated as 0.82 and 0.80, respectively (Figure 4g), demonstrating a mixed charge storage mechanism involving both diffusion controlled faradaic processes and surface capacitive contributions.

To further quantify these contributions of the MSC device, the current separation method was carried out by the following equation (Equation 2).

(2) $$i=k_{1}v+k_{2}v^{1/2}$$

where *k_1_v* represents the capacitive current contribution and *k_2_v^1/2^* accounts for the diffusion controlled faradaic component.^[^ ^36^ ^]^ As shown in Figure 4h, the surface capacitance contribution is 84% at 50 mV s^−1^, and the high capacitance of charge storage further demonstrates the excellent electrochemical properties of the porous MC2 electrode. In addition, the surface capacitance contribution of the MC2 electrode is higher than that of MC1, and MC3 (Figure 4i), demonstrating superior ion transport capacity at the MC2 electrode, and the high charge‐storage capacitance performance further proves its superior performance.

**Figure 5:**
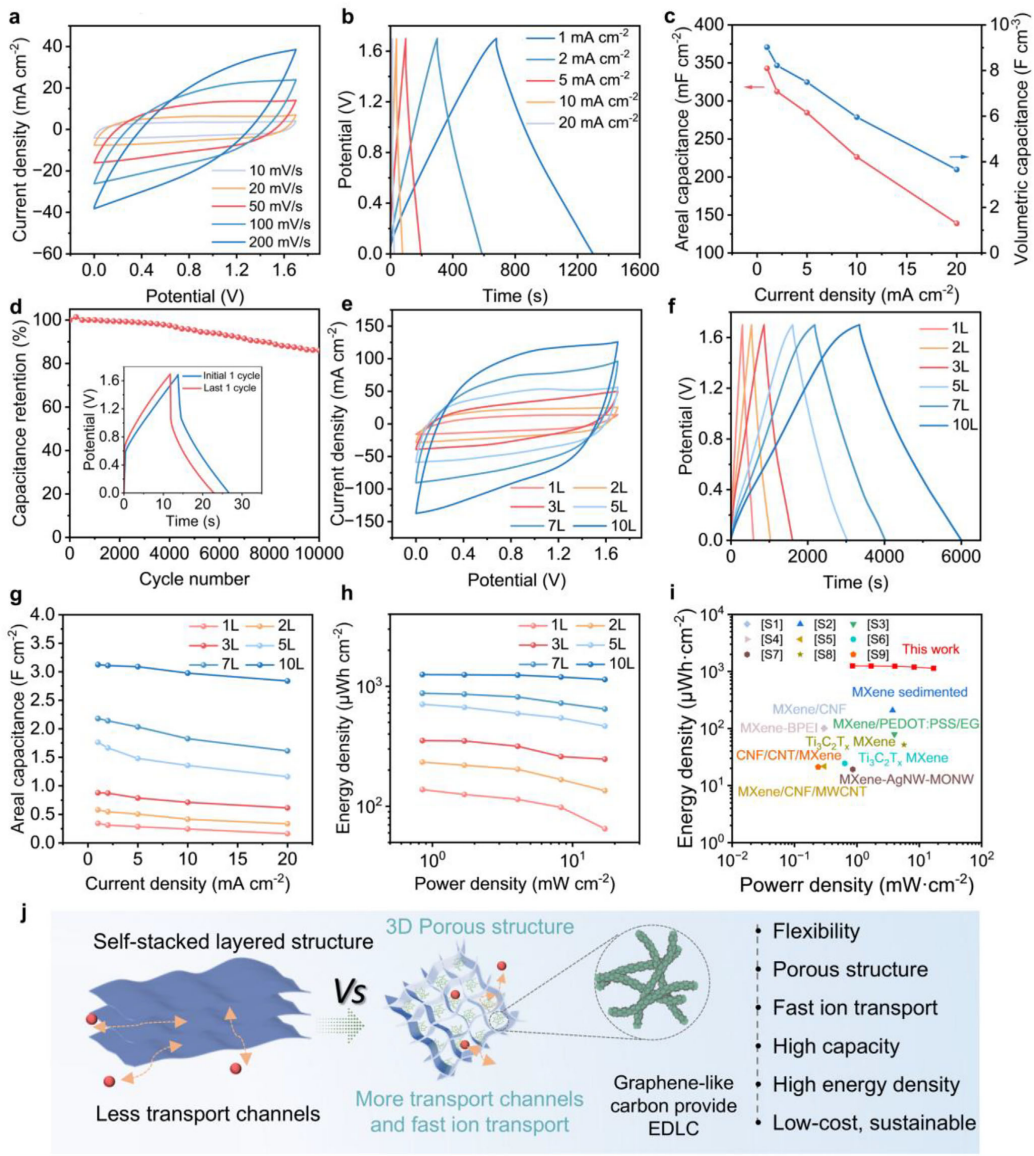
Electrochemical performances of MC2‐MSC: a) CV curves, b) GCD curves, c) areal and volumetric capacitances at different current densities, d) cycling performance at 20 mA cm^−2^ (Inset: GCD profile at first cycle and after 10 000 cycles). Electrochemical performances of MC2‐MSC with 3D printing various layers: e) CV curves (50 mV s^−1^), f) GCD curves at 2 mA cm^−2^, g) areal capacitances, h) Ragone plots, and i) Ragone plot of previously reported MXene‐based MSCs. j) Schematic diagram of the energy storage mechanism of the MC and MXene electrode.

The transition from aqueous electrolytes to all‐solid‐state configurations addresses critical limitations in operational voltage and thermal stability. The MC2‐MSC delivered exceptional areal (342.8 mF cm^−^^2^) and volumetric (9.02 F cm^−3^) capacitance at 1 mA cm^−^^2^, outperforming most reported MXene composites (**Figure** **5**c; Table S1, Supporting Information). This advancement stems from CC's graphene‐like morphology enhances EDLC formation while mitigating MXene restacking, as evidenced by comparative analyses with PH1000 and CNT additives (Figure S13, Supporting Information). Moreover, superior electrolyte‐electrode compatibility was confirmed through contact angle measurements (Figure S14, Supporting Information), indicating effective electrolyte permeation into the hierarchical pores of MC electrode.

Long‐term cycling tests revealed 86% capacitance retention after 10000 cycles at 20 mA cm^−^^2^ (Figure 5d), attributable to the stable 3D MC network that maintains structural integrity during repeated ion intercalation. Crucially, the graphene‐like coated cellulose layer on MXene not only maintains high conductivity while acting as a physical barrier, but also inhibits water/oxygen penetration to enhance electrode stability (Figure S15, Supporting Information). Capitalizing on the MC ink's precise thickness tunability (Figure S16, Supporting Information), we demonstrated thickness‐dependent performance scaling. All the CV and GCD curves of MC2‐MSC with different layers exhibit symmetrical shapes, conclusively affirming the swift and reversible characteristics of the electrode reactions (Figure 5e,f). The ten‐layer electrode achieved a specific capacitance value of 3.12 F cm^−^^2^, which represents a tenfold improvement in performance compared to single‐layer electrodes. Moreover, the capacitance exhibits a nearly linear correlation with thickness, confirming the depth independence of charge storage (Figure 5g; Figure S17, Supporting Information). The results indicate that by increasing the thickness of MC‐MSCs electrodes, a remarkable enhancement in both energy density and power density can be achieved simultaneously (Figure 5h). Critically, this scalability enables concurrent improvements in energy (1.25 mWh cm^−^^2^) and power densities (0.85 mW cm^−^^2^), surpassing state‐of‐the‐art symmetric supercapacitors in Ragone plot benchmarks (Figure 5i).

The exceptional electrochemical performance originates from a dual mechanism of interfacial synergy and structural optimization. The strategic integration of CC with MXene creates complementary charge storage pathways. The graphitic domains of CC enhance EDLC through rapid electron transport, while redox‐active surfaces of MXene contribute pseudocapacitance via efficient ion intercalation. This coupling effect is further amplified by the electrode's 3D hierarchical structure (Figure 5j), which features an interpenetrating porous framework that maximizes active site accessibility. The continuous MXene network embedded within the CC matrix facilitates unimpeded ion diffusion across the electrode depth, ensuring homogeneous charge distribution even under high current densities.

### 2.5 Mechanical Flexibility and Temperature Resilience of MC‐MSCs

**Figure 6:**
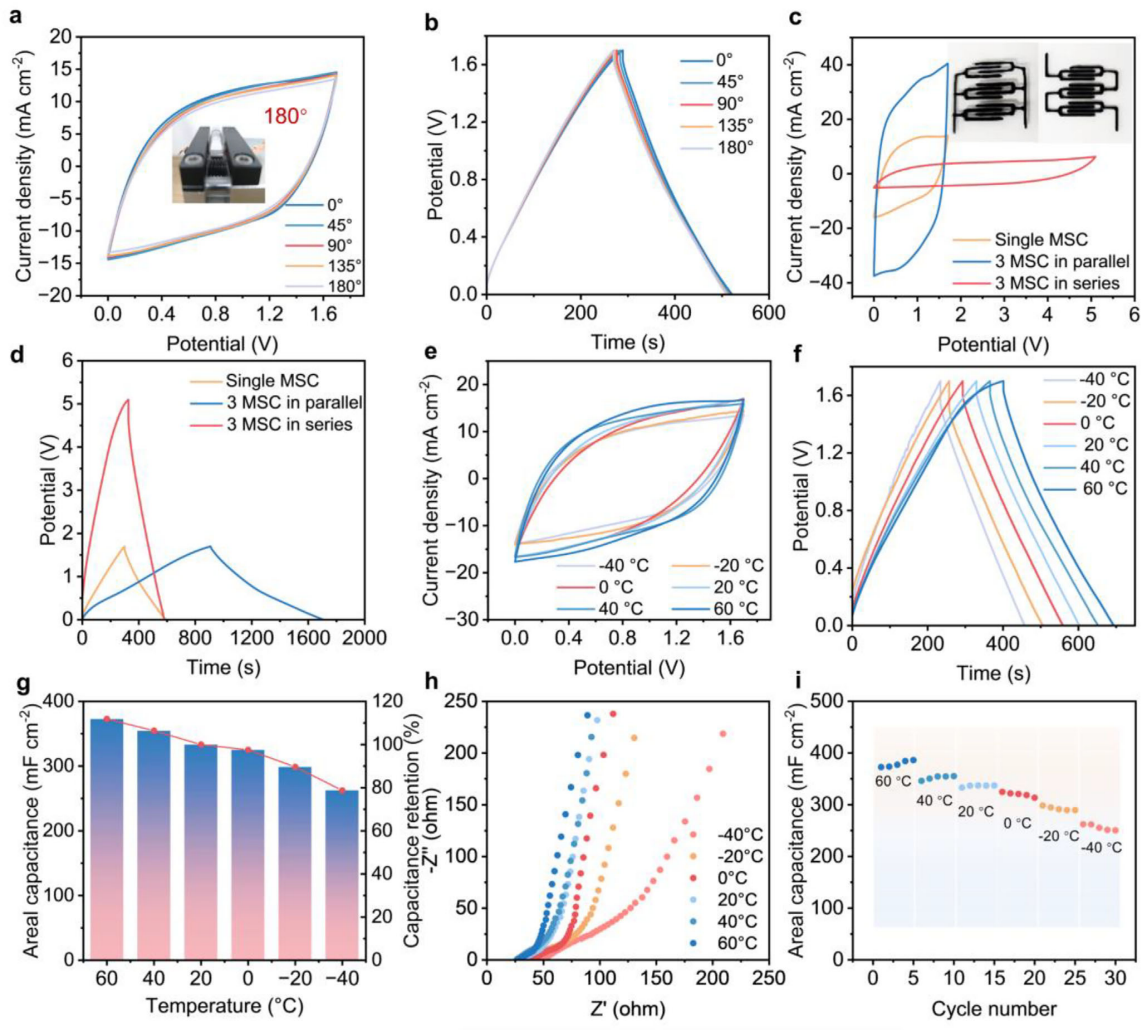
Mechanical flexibility, series and parallel integration, and temperature resilience of MC2‐MSCs. a) CV curves of MC2‐MSC under bending angles from 0° to 180°. b) Symmetric GCD curves across bending states, confirming mechanical integrity. c) Typical CV curves at 50 mV s^−1^ and d) GCD curves at 2 mA cm^−2^ for the integrated devices with three MC2‐MSC in series or parallel. e) CV curves and f) GCD curves of MC2‐MSC measured from −20 to 80 °C. g) Areal capacitances and capacitance retention, h) Nyquist plots, and i) Cycling stability of MC2‐MSC measured at different operating temperatures.

The exceptional bending durability of MC‐MSCs stems from CC's multifunctional design: its 3D entangled network concurrently suppresses MXene restacking and reinforces intermolecular interactions to maintain structural integrity during deformation, while interfacial hydrogen bonding between CC hydroxyl groups and MXene functionalities enables energy dissipation through controlled slip. Furthermore, CC‐imparted shear‐thinning rheology optimizes electrode printing uniformity, effectively mitigating stress concentration under cyclic bending, and collectively ensuring robust mechanical performance. This comprehensive mechanistic elaboration establishes explicit structure‐property relationships underlying the electrode's flexibility. As illustrated in **Figure** **6**a,b, the CV and GCD curves of MC2‐MSC nearly coincide during bending deformation. Furthermore, the printed MC2‐MSC device demonstrates remarkable deformation tolerance (Figure S18, Supporting Information). Even under significant bending angles of up to 180°, the MC2‐MSC retains ≈95% of its initial capacitance, showcasing its exceptional structural stability for flexible and wearable applications. Thanks to the exceptional rheological properties of the inks and the versatility of 3D printing in creating diverse shapes, we successfully fabricated integrated MC‐MSCs through a multi‐stage printing process. To further emphasize the customizability of MC2‐MSC, we constructed various configurations of MC2‐MSCs connected in series and parallel (Figure S19, Supporting Information). As evident from the CV curves and GCD profiles (Figure 6c,d), the total current output and discharge time of the integrated MC2‐MSC increased proportionally with the number of parallel‐connected MSCs, leading to a linear enhancement in capacitance. Meanwhile, the MC2‐MSCs connected in series exhibited a step‐wise increase in voltage, from 1.7 V for a single cell to 5.4 V for three MSCs.

To showcase its superior performance in harsh environmental conditions, we thoroughly assessed the electrochemical performance of MC2‐MSC across a range of extreme environmental settings (Figure 6e‐i). Owing to the low freezing point and heat resistance of the LiCl/SiO_2_ gel electrolytes, MC2‐MSC demonstrated robust operation over a broad temperature range of −40 to 60 °C. We observed a notable increase in both the capacitance and rate performance of MC2‐MSC with rising temperatures (Figure 6f,g). However, at −40 °C, a significant decrease in capacitance was observed. Figure 6g illustrates that MC2‐−MSC retained nearly 111% and 89% of its original capacitance at 60 °C and −20 °C, respectively, attributed to the superior high‐temperature and frost resistance of the LiCl/SiO_2_ gel electrolyte. Additionally, the Nyquist plot of MC2‐MSC at temperatures ranging from −40 °C to 60 °C revealed that impedance and ion diffusion resistance decreased with increasing temperature, while they gradually increased with decreasing temperature (Figure 6h). Notably, at −40 °C, the GCD curve still exhibited a high capacitance of 262.3 mF cm^−2^ at 2 mA cm^−2^, corresponding to a high capacitance retention of 79% compared to 20 °C (Figure 6i). Such low temperature tolerance of high concentration LiCl and the high compatibility with microelectrodes ensure that MSCs with excellent electrochemical performance retained high capacitance at low temperatures. The high compatibility of this low temperature tolerant electrolyte with the MC‐electrodes ensures that MC‐MSCs maintain high capacitance at low temperatures. Moreover, after undergoing 10000 cycles at a current density of 20 mA cm^−2^, they still retain nearly 83% of their capacitance (Figure S20, Supporting Information), showcasing exceptional durability and low‐temperature performance. These results indicate that MC‐MSCs have a promising application in cold conditions.

### 2.6 All‐In‐One Self‐Powered Integrated System

**Figure 7:**
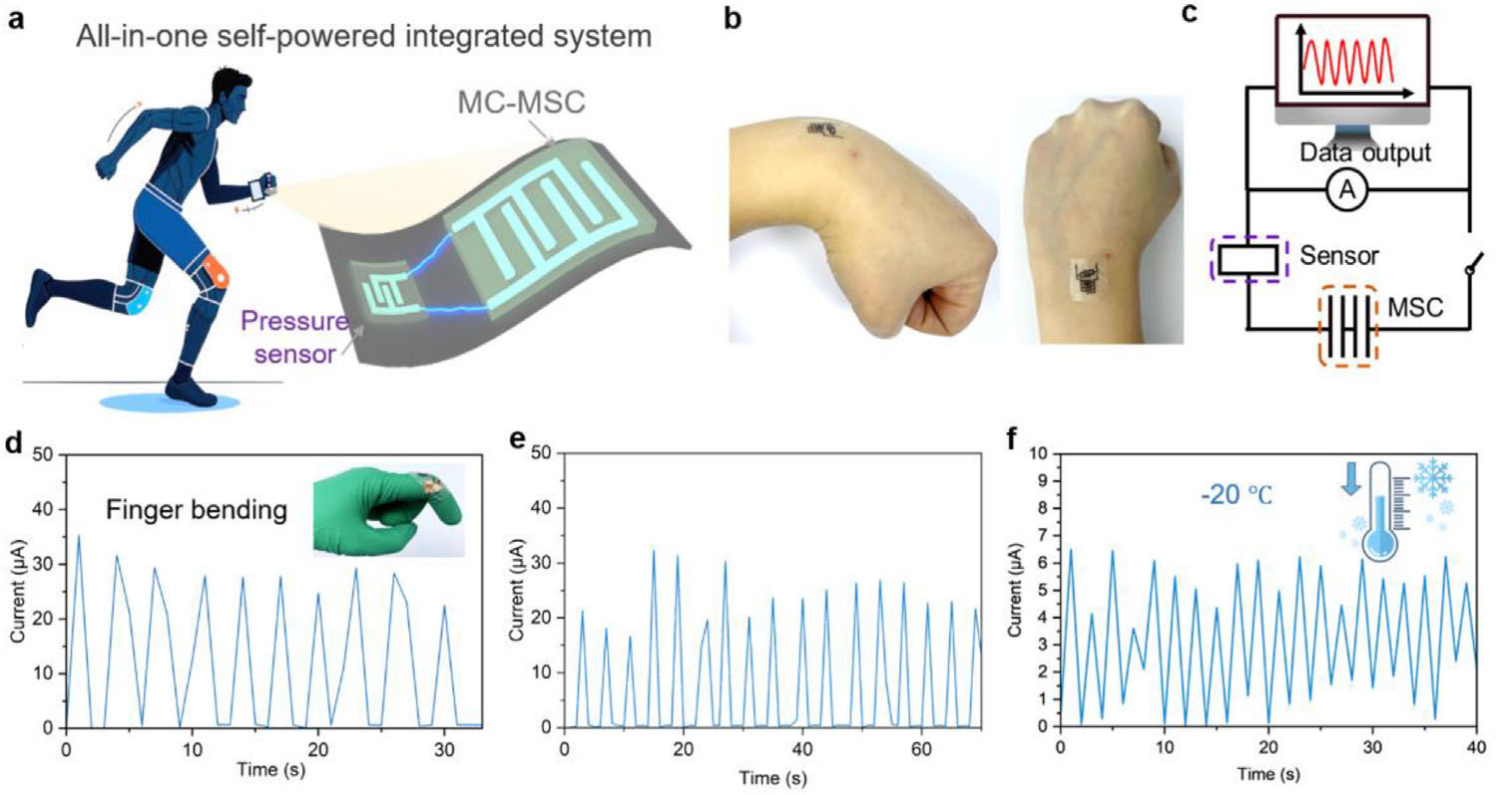
All‐in‐one self‐powered integrated system for wearable biomechanical monitoring. a) Schematic illustration of the integrated system comprising 3D printed MC2‐MSCs and strain sensors for energy storage and motion detection. b) Photographs of microelectrodes of 3D printed MC2‐MSC sensor integrated system. c) Equivalent circuit diagram of the integrated system. Real‐time current responses to d) finger bending and e) wrist flexion. f) Current signals during finger bending at −20 °C.

The convergence of energy storage and sensing functionalities was achieved through the rational design of MC hierarchical architectures. We developed an autonomous self‐powered system by monolithically integrating series‐connected MC‐MSCs with strain sensors using multifunctional MC2 ink (**Figure** **7**a,b). The MC2 ink served dual functions as both a high‐performance electrode material and strain‐sensing component. Specifically, the MSC powered strain sensor generated measurable electrical signals in response to bodily motions, which were then transmitted via a USB enabled multimeter and displayed on a computer interface for immediate analysis (Figure 7c). The integrated sensor stably measured the weak signal caused by the bending of the wrist and fingers (Figure 7d,e; Figure S21, Supporting Information). And can maintain excellent signal transmission in low temperature environments (Figure 7f).

## 3 Conclusion

In summary, we demonstrated fully 3D printed flexible MSCs with outstanding areal/volumetric capacity and rate capability using a thixotropic electronic ink comprising 1D conductive cellulose and 2D Ti_3_C_2_T*_x_* MXene nanosheets. Attributable to synergistic interactions between its constituent materials and optimized electrode architecture, the 3D printed MSC device achieved unprecedented electrochemical performance, including a landmark areal capacitance of 3.12 F cm^−2^, outstanding energy density of 1.25 mWh cm^−2^, and 94% capacitance retention after 10 000 bending cycles. In addition, an all‐in‐one integrated sensing system powered by the 3D printed MSC was also found to be capable of monitoring human motion. Consequently, our approach may serve as a viable reference for the scalable fabrication of components that are easily integrated, self‐sustained, and customizable for the next generation of integrated, wearable, and implantable microelectronics. This work may open up a new avenue for the fabrication of integrated, wearable, and implantable microelectronics.

## Conflict of Interest

The authors declare no conflict of interest.

## Supporting information



Supporting Information

## Data Availability

The data that support the findings of this study are available from the corresponding author upon reasonable request.

## References

[1] X. Q. Mi , L. X. Liu , S. J. Yang , P. Q. Wu , W. Q. Zhan , X. Y. Ji , J. J. Liang , Nat. Commun. 2025, 16, 2590. 40091079 10.1038/s41467-025-57959-4 PMC11911445

[2] X. Y. Li , X. J. Luo , D. S. Chen , L. X. Yang , H. Wang , T. Gao , Y. S. Liu , J. Lin , Adv. Mater. 2025, 37, 2417763. 10.1002/adma.202417763 40317541

[3] S. T. Cheng , Z. H. Zhang , J. F. Yan , T. Y. Yang , J. L. Zhang , J. C. Fu , Adv. Funct. Mater. 2025, 10.1002/adfm.202502526.

[4] Z. Q. Cao , Y. B. Zhu , K. Chen , Q. Wang , Y. J. Li , X. J. Xing , J. Ru , L. G. Meng , J. Shu , N. Shpigel , L. F. Chen , Adv. Mater. 2024, 36, 2401271. 10.1002/adma.202401271 38549262

[5] F. Leng , M. Zheng , C. Xu , Exploration 2021, 1, 20210109. 37323692 10.1002/EXP.20210109 PMC10190842

[6] H. P. Li , S. M. Ding , J. B. Ding , J. H. Luo , S. R. Liu , H. B. Huang , Energy Storage Mater. 2025, 74, 103907.

[7] H. C. Ye , Y. He , T. T. You , F. Xu , Adv. Funct. Mater. 2025, 35, 2413343.

[8] S. Nouseen , M. Pumera , Adv. Funct. Mater. 2025, 35, 2421987.

[9] S. Sahoo , R. Kumar , I. Hussain , K. Zhang , Adv. Powder Mater. 2024, 3, 100246.

[10] Y. L. Zhou , J. Li , H. Y. Fu , N. Li , S. M. Chai , T. F. Duan , L. J. Xu , Z. J. Wang , J. X. Xu , Carbon Energy 2025, 7, 698.

[11] C. K. Maity , S. De , A. De Adhikari , A. Kumari , K. Verma , M. Moniruzzaman , S. Sahoo , Energy Storage Mater. 2024, 73, 103873.

[12] Z. Wu , S. Liu , Z. Hao , X. Liu , Adv. Sci. 2023, 10, 2207174. 10.1002/advs.202207174 PMC10323642 37096843

[13] M. A. Nazir , T. Najam , S. Ullah , I. Hossain , M. S. Javed , M. Naseer , A. u. Rehman , S. S. A. Shah , EcoEnergy 2024, 2, 505.

[14] L. F. Chen , Y. Feng , H. W. Liang , Z. Y. Wu , S. H. Yu , Adv. Energy Mater. 2017, 7, 1700826.

[15] G. Shi , Y. X. Zhu , M. Batmunkh , M. Ingram , Y. F. Huang , Z. H. Chen , Y. J. Wei , L. X. Zhong , X. W. Peng , Y. L. Zhong , ACS Nano 2022, 16, 14723. 36001805 10.1021/acsnano.2c05445

[16] G. Q. Zhou , M. C. Li , C. Z. Liu , Q. L. Wu , C. T. Mei , Adv. Funct. Mater. 2022, 32, 2109593.

[17] J. Ding , Q. Wang , X. Liu , S. Li , H. Li , J. Hazard. Mater. 2024, 480, 136261. 39447231 10.1016/j.jhazmat.2024.136261

[18] K. M. Wyss , D. X. Luong , J. M. Tour , Adv. Mater. 2022, 34, 2106970. 10.1002/adma.202106970 34695282

[19] M. Mayyas , H. Z. Li , P. Kumar , M. B. Ghasemian , J. Yang , Y. F. Wang , D. J. Lawes , J. L. Han , M. G. Saborio , J. B. Tang , R. Jalili , S. H. Lee , W. K. Seong , S. P. Russo , D. Esrafilzadeh , T. Daeneke , R. B. Kaner , R. S. Ruoff , K. Kalantar‐Zadeh , Adv. Mater. 2020, 32, 2001997. 10.1002/adma.202001997 32510699

[20] D. C. Wang , H. Y. Yu , D. M. Qi , Y. H. Wu , L. M. Chen , Z. H. Li , J. Am. Chem. Soc. 2021, 143, 11620. 34286968 10.1021/jacs.1c04710

[21] M. Matsumoto , Y. Saito , C. Park , T. Fukushima , T. Aida , Nat. Chem. 2015, 7, 730. 26291945 10.1038/nchem.2315

[22] J. Overbeck , G. B. Barin , C. Daniels , M. L. Perrin , O. Braun , Q. Sun , R. Darawish , M. De Luca , X. Y. Wang , T. Dumslaff , A. Narita , K. Müllen , P. Ruffieux , V. Meunier , R. Fasel , M. Calame , ACS Nano 2019, 13, 13083. 31573799 10.1021/acsnano.9b05817

[23] W. Chen , Y. Liu , X. Zhang , M. Liu , D. Han , X. Song , L. Tan , X. Wu , J. Power Sources 2025, 654, 237814.

[24] G. A. Rance , D. H. Marsh , R. J. Nicholas , A. N. Khlobystov , Chem. Phys. Lett. 2010, 493, 19.

[25] H. Li , S. Liu , X. Li , Z.‐S. Wu , J. Liang , Mater. Chem. Front. 2019, 3, 626.

[26] H. Li , J. Liang , Adv. Mater. 2020, 32, 1805864. 10.1002/adma.201805864 30941808

[27] A. Asghar , M. S. Rashid , M. Hanif , I. Boukhris , Z. W. Chen , M. Saqib , Q. Arshad , P. Rosaiah , S. Ali , I. Hussain , Chem. Eng. J. 2025, 506, 159811.

[28] D. X. Cao , Y. J. Xing , K. Tantratian , X. Wang , Y. Ma , A. Mukhopadhyay , Z. Cheng , Q. Zhang , Y. C. Jiao , L. Chen , H. L. Zhu , Adv. Mater. 2019, 31, 1807313. 10.1002/adma.201807313 30761614

[29] L. Li , J. Meng , X. R. Bao , Y. P. Huang , X. P. Yan , H. L. Qian , C. Zhang , T. X. Liu , Adv. Energy Mater. 2023, 13, 2203683.

[30] L. Li , Y. Zhang , H. Y. Lu , Y. F. Wang , J. S. Xu , J. X. Zhu , C. Zhang , T. X. Liu , Nat. Commun. 2020, 11, 62. 31911636 10.1038/s41467-019-13959-9 PMC6946679

[31] J. Wen , Z. Song , J. Ding , F. Wang , H. Li , J. Xu , C. Zhang , J. Mater. Sci. Technol. 2022, 114, 233.

[32] S. J. Guan , Y. Yang , Y. Y. Wang , X. Zhu , D. D. Ye , R. Chen , Q. Liao , Adv. Mater. 2024, 36, 2305854. 10.1002/adma.202305854 37671789

[33] H.‐P. Li , J. Wen , S.‐M. Ding , J.‐B. Ding , Z.‐H. Song , C. Zhang , Z. Ge , X. Liu , R.‐Z. Zhao , F.‐C. Li , Nano Mater. Sci. 2023, 5, 421.

[34] H. Li , X. Li , J. Liang , Y. Chen , Adv. Energy Mater. 2019, 9, 1803987.

[35] L. Liu , Z. Du , J. Wang , H. Du , S. Wu , M. Li , Y. Zhang , J. Sun , Z. Sun , W. Ai , Research 2023, 6, 0209. 37593340 10.34133/research.0209 PMC10430870

[36] X. Shi , X. Liu , E. Wang , X. Cao , Y. Yu , X. Cheng , X. Lu , Carbon Neutraliz. 2023, 2, 28.

