# Supplementary material for: Sustainable MXene/Conductive Cellulose Heteroinks for 3D Printed High Areal Energy Density Micro‐Supercapacitors and Self‐Powered Integrated Systems
Source: Adv Sci (Weinh). 2025 Aug 12;12(41):e11439. doi: 10.1002/advs.202511439 (PMC12591215; doi:10.1002/advs.202511439)
Supplement: Supplementary file 1 — Supporting Information [file ADVS-12-e11439-s001.docx]

Supplementary Information

**Sustainable MXene/Conductive Cellulose Heteroinks for 3D Printed High Areal Energy Density Micro-Supercapacitors and Self-Powered Integrated Systems**

*Chunling Cao, Shiyao Tang, Xiaofei Wu, Shouxin Liu*, Haibo Huang*, Hongpeng Li**

C. Cao, S. Tang, X. Wu, Prof. S. Liu, Prof. H. Huang

Key Laboratory of Bio-Based Material Science and Technology (Ministry of Education), Northeast Forestry University, Harbin 150040, China

Email: [supernova_bo@nefu.edu.cn](mailto:supernova_bo@nefu.edu.cn) (H. Huang), [liushouxin@126.com](mailto:liushouxin@126.com) (S. Liu)

Prof. H. Li

College of Mechanical Engineering, Yangzhou University, Yangzhou 225127, China

School of Automotive Engineering, Nantong Institute of Technology, Nantong 226001, China

Email: [lihongpeng@yzu.edu.cn](mailto:lihongpeng@yzu.edu.cn) (H. Li)

**Experimental Section**

***Synthesis of Ti_3_C_2_T_x_ (MXene) nanosheets***

Typically, 1.2 g LiF and 20 mL HCl (9 M) were mixed under magnetic stirring at room temperature for 30 min in a polytetrafluoroethylene (PTFE) container to form a homogeneous solution. Then, 1 g Ti_3_AlC_2_ powder was slowly added to the above solution, followed by stirring at 40℃ for 36 h to remove the aluminum layer completely. The resulting suspension was washed with deionized (DI) water and centrifuged (3500 rpm, 5 min) for several times until the pH was close to neutral. After dispersing multilayer Ti_3_C_2_T_x_ was exfoliated to Ti_3_C_2_T_x_ nanosheet in DI water by sonicating under argon flow for 30 min. Finally, the solution was centrifuged at 3500 rpm for 1 h, and the black and green supernatant containing the Ti_3_C_2_T_x_ flakes was collected.

***Synthesis of conductive cellulose***

Typically, 1 g of bamboo powder was placed into a three-neck flask and slowly added 80 mL of 64 wt% H_2_SO_4_ (the temperature of H_2_SO_4_ should not be higher than 25℃), sealed completely with a rubber plug, and passed high-flux nitrogen for 30 minutes. After that, it was hydrolyzed at 40℃ for 30 minutes. Self-limiting carbonization of CNF nanosurface was directly heated at 90℃ and rapid stirring for 1h, 2h, and 4h. The whole process was sealed and protected by high-flux nitrogen. At the end of the reaction, the suspension is quickly cooled to room temperature in a cold bath and dialyzed to neutral. The samples were named CC1, CC2, and CC4 according to the reaction time.

***Preparation of MXene/CC ink***

CC1 powder was added into MXene suspension (10 mg/ml) under continuous stirring for 2 h to form homogeneous hybrid dispersions. The solid content ratios of CC1 to MXene were 9:1, 8:2, and 7:3. Subsequently, the suspension was concentrated via super absorbent polymer (SAP) beads to obtain the printable composite ink with proper viscosity and rheological properties, and named respectively as MC1, MC2, MC3. And the 8:2 ratios of CC2 and CC4 to MXene were named MCC2 and MCC4.

***Synthesis of gel electrolyte***

LiCl/SiO_2_ gel electrolyte: First, 20 mol LiCl salt was completely dissolved in 1 L deionized water at room temperature and denoted as a 20 m LiCl electrolyte. Then, approximately 6 wt% SiO_2_ powder (Sigma-Aldrich) was added and mixed evenly to form a transparent gel, denoted as a 20 m LiCl-gel electrolyte.

***Preparation of 3D Printed MSCs***

The 3D printing process of MC ink was carried out using an MP1100 3D bioprinter (Shanghai Mifang Electronic Technology Co., Ltd., China) equipped with a pneumatic dispenser. The prepared printable ink was loaded into a syringe and assembled with a needle (diameter = 0.41 mm), which was then connected to an air-controlled dispenser to adjust the printing pressure. Extruded layer by layer on a polyethylene terephthalate (PET) substrate. Using the printing method described above (printing pressure 50 kPa, printing speed: 1 mm s^-1^, needle diameter: 410 µm), the printed MXene/CC gel structures were frozen at -4℃ and then freeze-dried for 48 hours to obtain self-supporting 3D structures. Subsequently, the prepared LiCl/SiO_2_ gel electrolyte was cast onto the interdigital electrodes. Last, the prepared encapsulation solution was directly coated onto the whole device, followed by natural drying at room temperature in a fume hood to obtain the assembled MSCs.

***Assembly of the self-powered integrated sensing systems***

Firstly, interdigitated electrodes and pressure sensors are 3D printed onto a PET substrate. Subsequently, the structure is freeze-dried and then interconnected using conductive silver paste as the conductive interconnect. LiCl/SiO_2_ electrolyte is then dispensed onto it. Finally, the entire system is encapsulated with Ecoflex to obtain a self-powered integrated sensing system. The wireless display in a mobile phone (or computer) app in real-time was achieved through a wireless meter with a built-in Bluetooth transmission module (UT61E+, purchased from Yuride Technology (China) Co., LTD).

***Structural characterization***

The morphology and structure of the samples were characterized by X-ray diffractometer (XRD, Shimatsu XRD-6100, Japan), scanning electron microscopy (SEM, JSM-7500F), transmission electron microscopy (TEM, Talos F200s), atomic force microscope (AFM, Multimode 8), X-ray photoelectron spectroscopy (XPS, Thermo Scientific K-ALPHA). Raman spectra were characterized by Raman spectrometer (J-Y, HR800, France) under an excitation wavelength of 532 nm.

The rheological properties of the inks were probed using a DHR-2 rheometer with a 20 mm parallel plate geometry and a 1 mm gap. All measurements were taken at 25 °C. The steady shearing measurement was conducted at the shear rates from 0.01 to 100 s^-1^. The extrusion printing process was simulated by recording the change of viscosity with time when the shear rate changed periodically, and the procedure was programmed as follows: 0.01 s^-1^ shear rate for 60 s, 100 s^-1^shear rates for 60 s, 0.01 s^-1^ shear rate for 150 s. The oscillatory strain sweep experiments were performed at a frequency of 1 Hz to characterize the storage modulus (G′) and the loss modulus (G″) of the ink.

***Electrochemical characterization***

The electrochemical characterization of a single electrode was performed in a conventional three-electrode setup using 1 M H_2_SO_4_ solution as the electrolyte, a Pt electrode as the counter electrode, and an Ag/AgCl electrode as the reference electrode. The carbon paper was used as the current collector, and a working electrode was prepared by mixing 80 wt% printing ink, 10 wt% carbon black as the conductive additive, and 10 wt% polytetrafluoroethylene (PTFE) binder (geometric area and mass loading of ~1 cm^-2^ and ~5 mg cm^-2^, respectively). For the assembly and performance testing of MSCs, we used the conductive Cu adhesive tape (SPI SUPPLIES) to connect microelectrode fingers with the external circuit, which was linked to the electrochemical workstation (CHI760E) for measurement. Cyclic voltammetry (CV) tests were performed in the potential range from 0 to 1.7 V under the scan rate from 10 to 200 mV s^-1^. Galvanostatic charge-discharge (GCD) tests were performed over a voltage range from 0 to 1.7 V at a current density from 1 to 20 mA cm^-1^. Electrochemical impedance spectroscopy (EIS) was conducted in a frequency range from 0.01 to 10^5^ Hz with a 5 mV amplitude at an open-circuit potential.

The dimensions of the 3D printed MC block architecture are geometric area: 0.2 cm^2^; height: 0.38 mm. Detailed calculation methods of specific capacitance C_g_ (F g^-1^), areal capacitance C_s_ (mF cm^-2^), volumetric capacitances C_v_ (F cm^-3^), energy density E (μWh cm^-2^) and power density P (μW cm^-2^) are based on Equations (1)-(5).

*C_g_=(I×t)/(∆U×m)* (1)

*C_s_=(I×t)/(∆U×s)* (2)

*C_v_=(I×t)/(∆U×V)* (3)

Where *I* (A) stands for discharge current; *t* (s) represents discharge time; *∆U* (V) is the voltage range during discharging; *m* (g) is the mass loading of active material, *s* (cm^-2^) is the total area of the device, and *V* (cm^-3^) is the total volume of the device.

*E=(1/2)×(1/3.6)×C×(∆U)^2^*  (4)

*P=3600×E/t*  (5)

Where E (Wh kg^-1^, μWh cm^-2^, Wh L^-1^) and P (W kg^-1^, μW cm^-2^, W L^-1^) are the energy density and power density of the MSCs, respectively. C (F·g^-1^, mF·cm^-2^, F·cm^-3^) is the capacitance calculated from Equation (1)-(3).


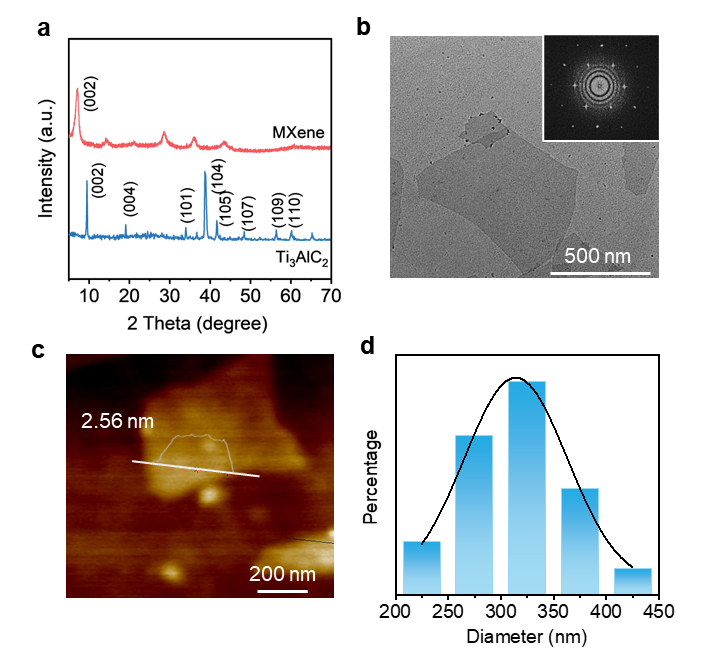


**Figure S1**. a) XRD pattern, b) TEM image (Inset: selected-area electron diffraction pattern), and c) AFM image of MXene nanosheets. d) Lateral size distribution of MXene nanosheets.


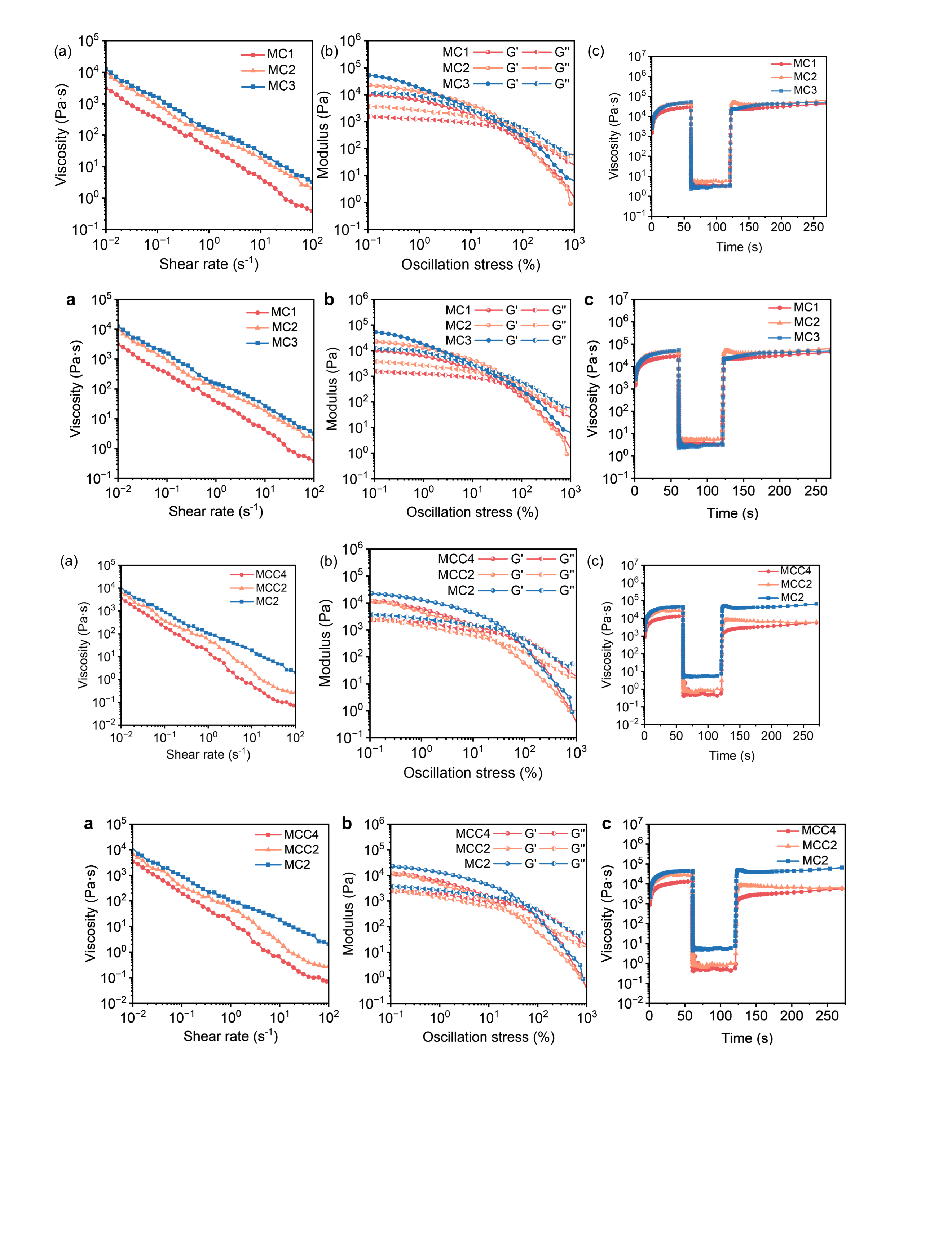


**Figure S2.** Rheological characterization of 3D printable MC inks with different ratios. a) Relationship between the viscosity of printing ink and shear rate. b) The relationship between storage modulus (G') and loss modulus (G'') of oscillatory stress. c) The viscosity versus time curve for simulating the ink extrusion process in 3D printing.


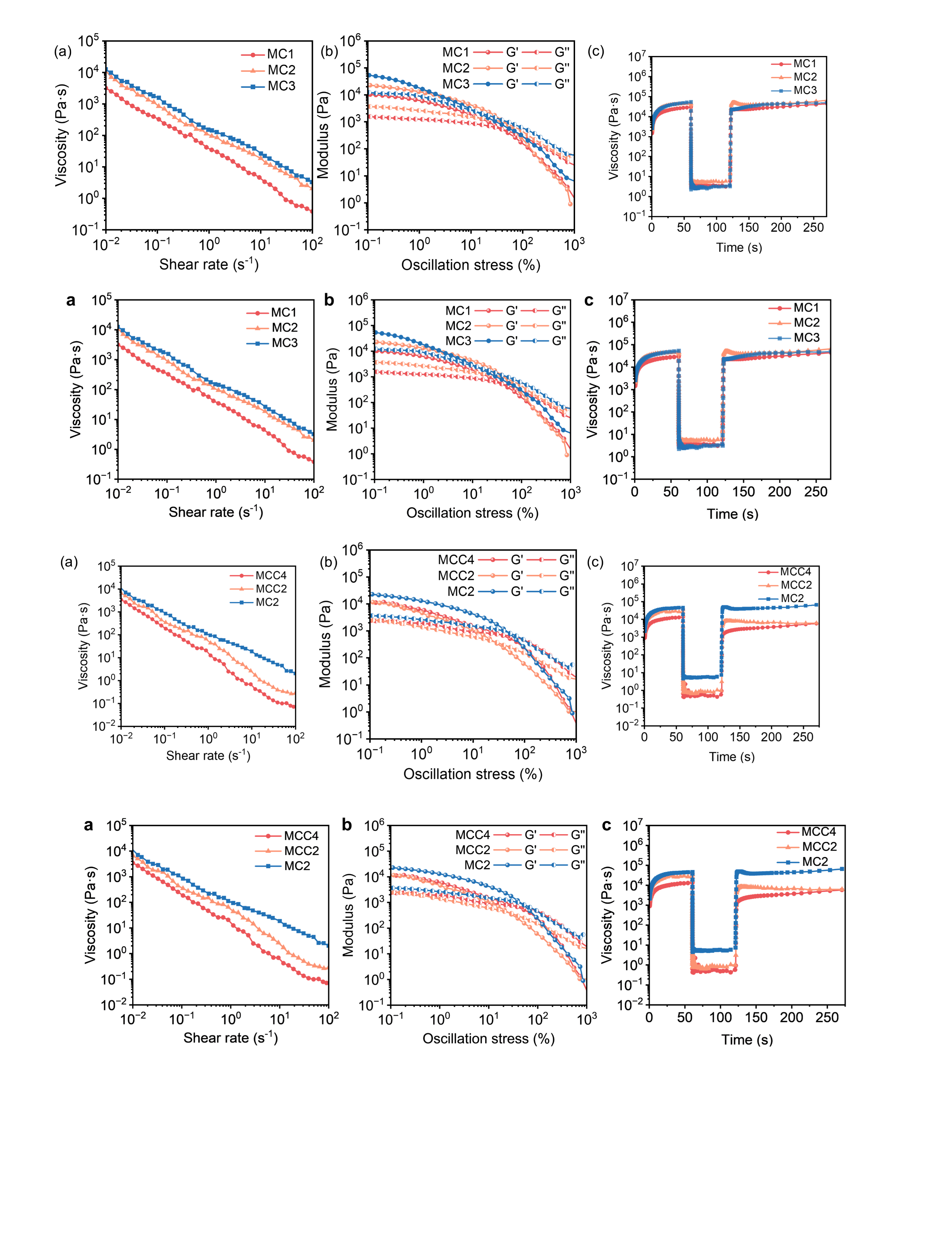


**Figure S3.** Rheological characterization of 3D printable MC inks with CC different reaction times. a) Relationship between the viscosity of printing ink and shear rate. b) The relationship between storage modulus (G') and loss modulus (G'') of oscillatory stress. c) The viscosity versus time curve for simulating the ink extrusion process in 3D printing.


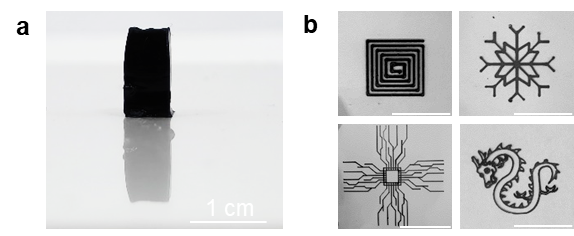


**Figure S4.** a) Photograph of a freestanding 3D-printed multilayer architecture showcasing structural integrity. b) Photograph of diverse high-fidelity geometric patterns printed using MC inks, scale bar: 10 mm.


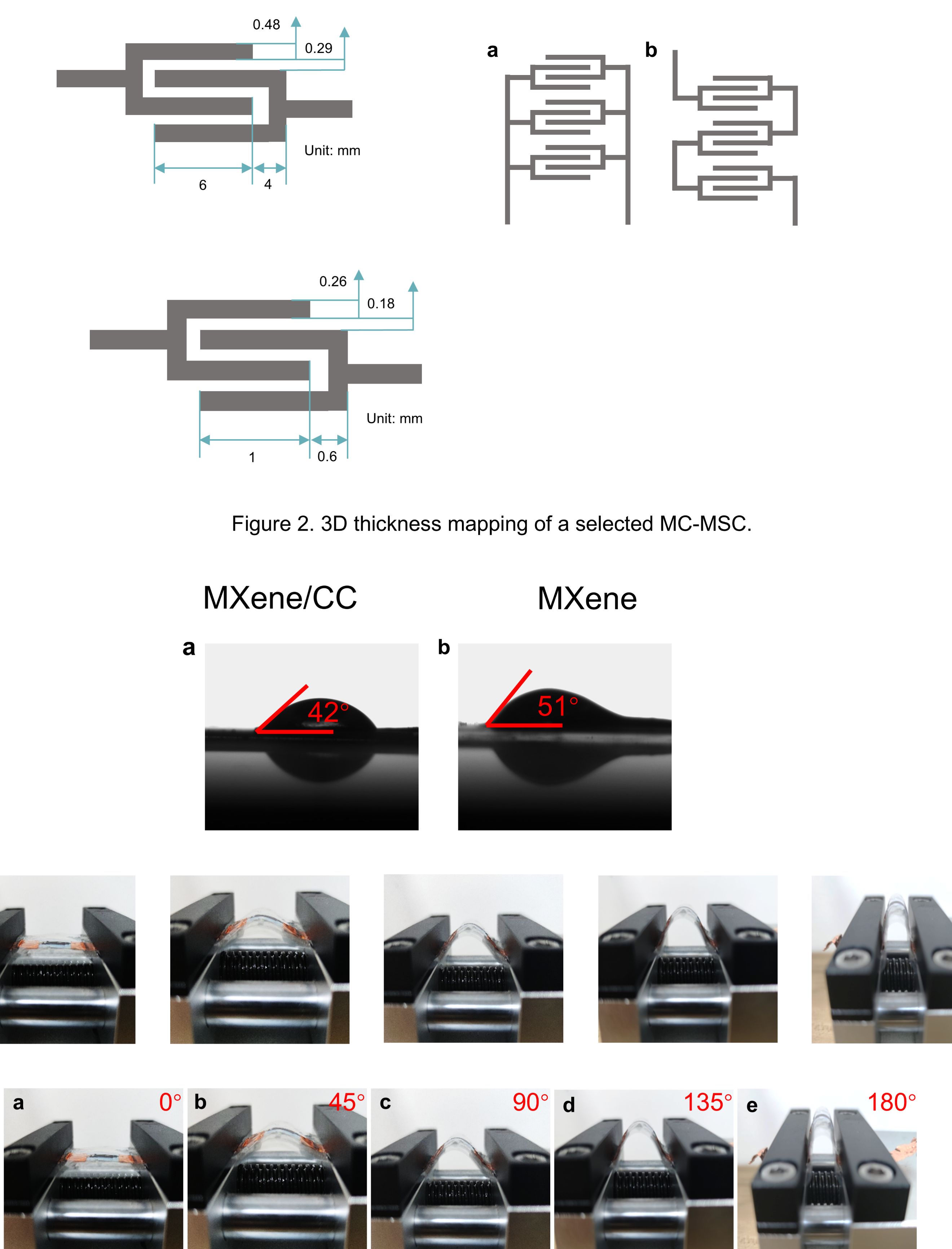


**Figure S5.** Schematic illustration of the microelectrode size parameters.


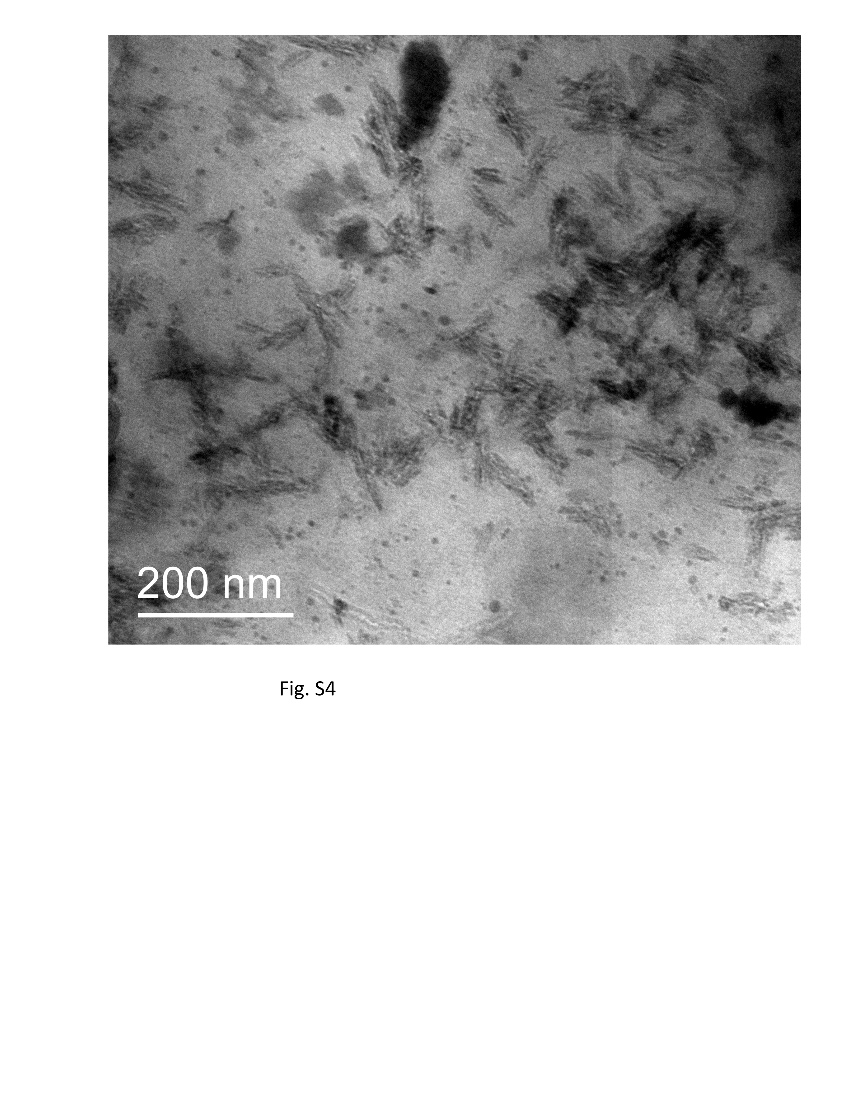


**Figure S6.** TEM images of MC2 gel composites.


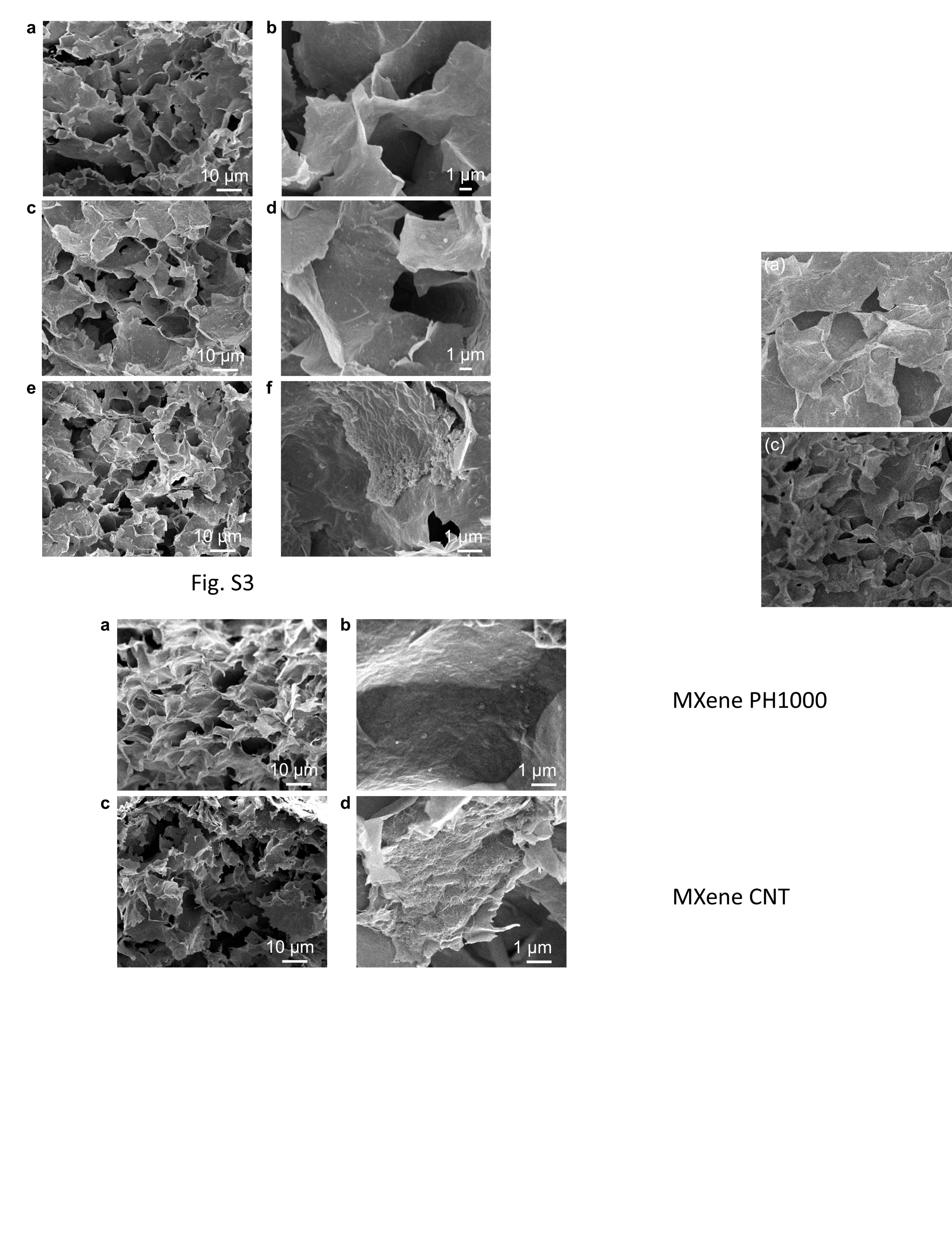


**Figure S7.** SEM images of (a, b) MXene, (c, d) MC1, and (e, f) MC3 electrode, respectively.


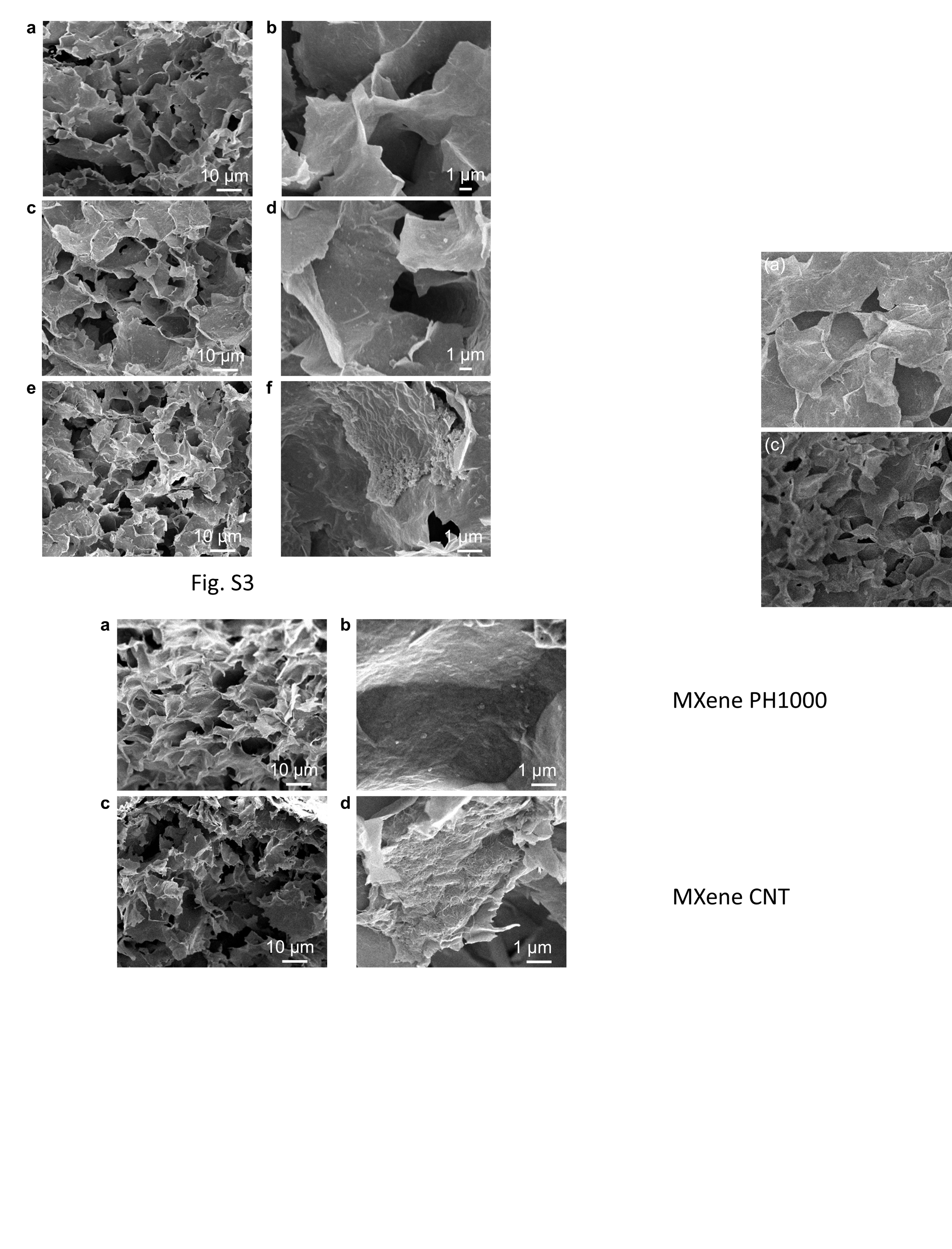


**Figure S8.** a, b) SEM images of the MXene/PH1000 electrode at low and high magnifications, respectively. c, d) SEM images of the MXene/CNT electrode at low and high magnifications, respectively.


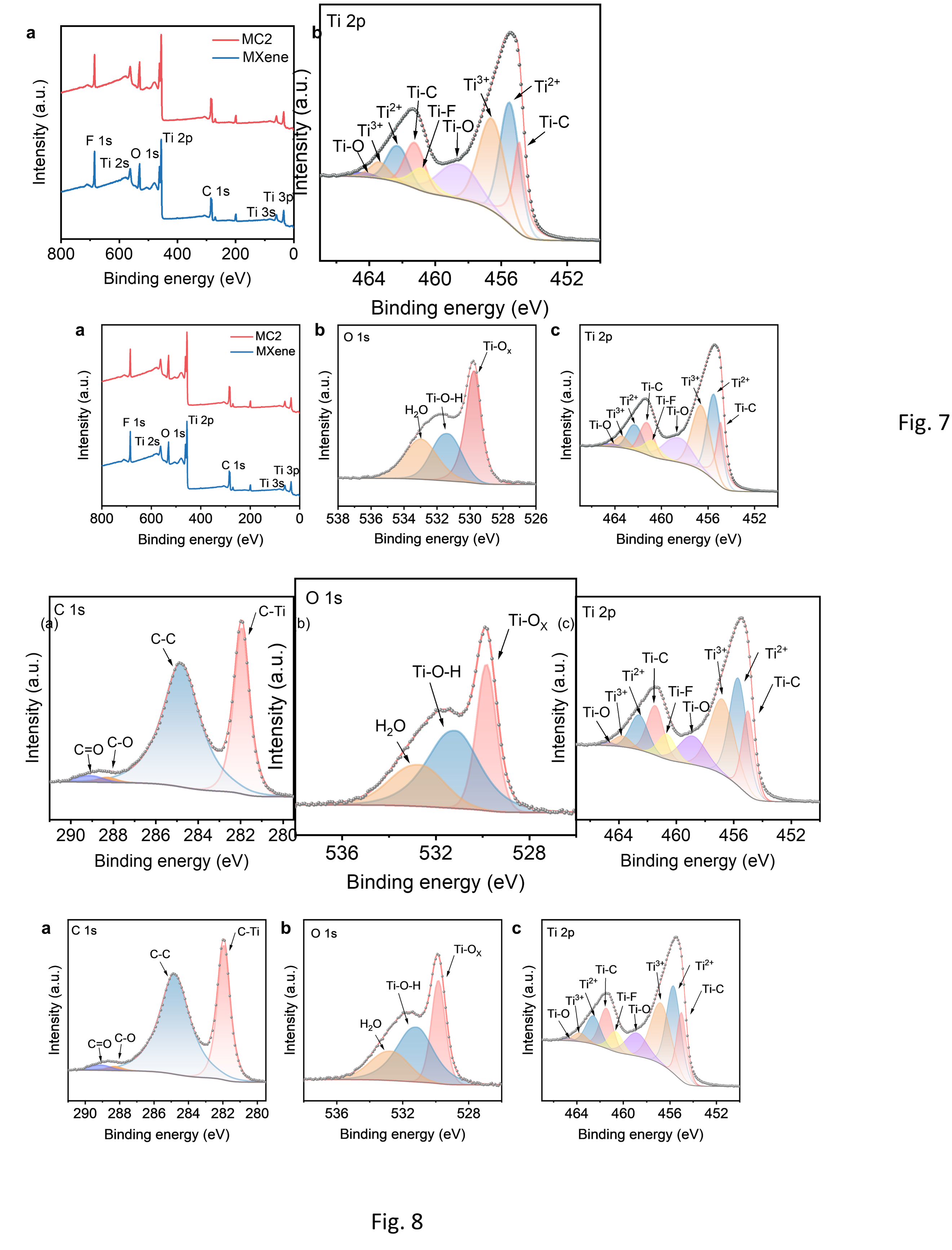


**Figure S9.** XPS spectra of MC2 electrode material. a) Full survey spectra. High-resolution b) O 1s spectra. c) High-resolution Ti 2p spectra.


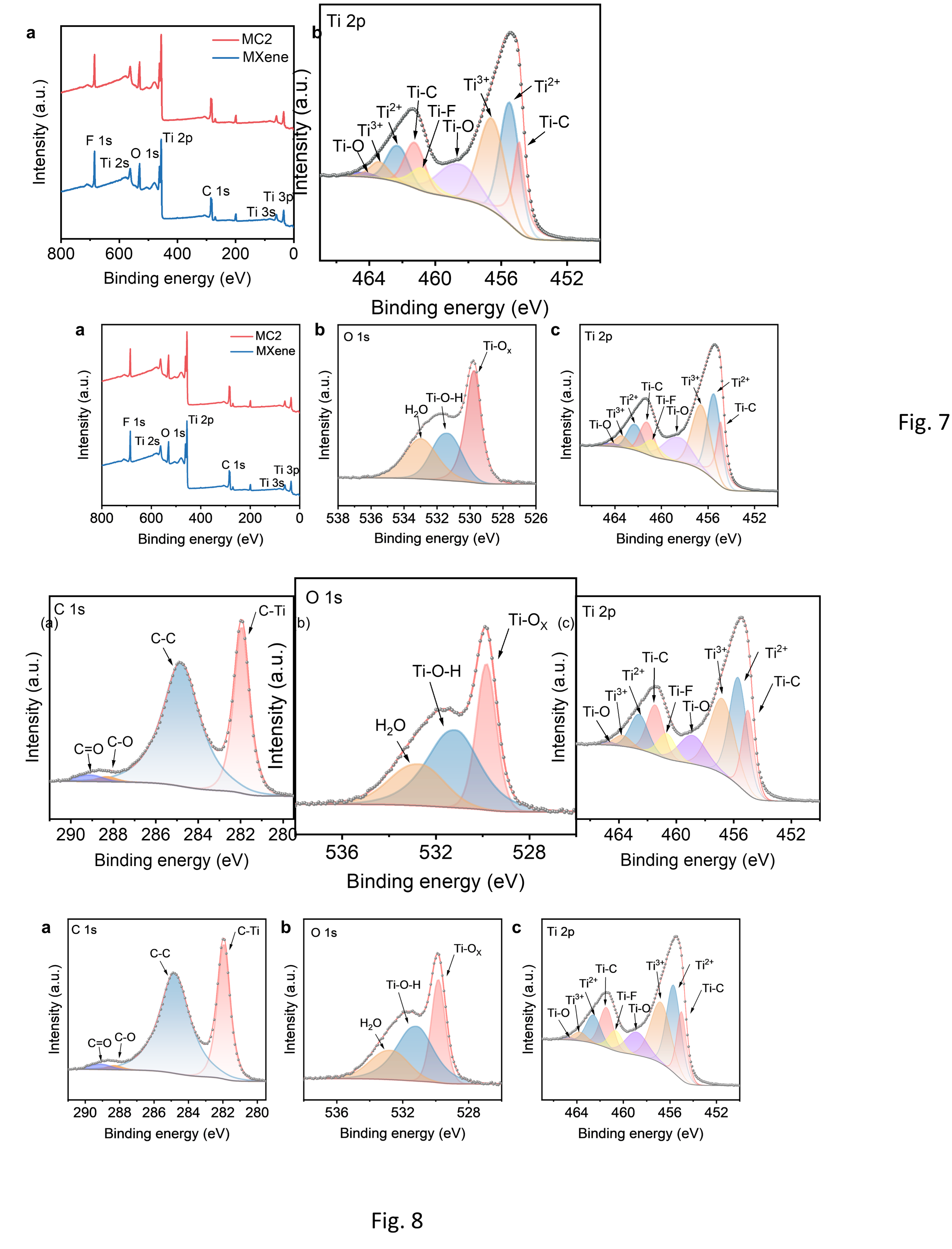


**Figure S10.** XPS spectra of MXene electrode material. a) High-resolution C 1s spectra. b) High-resolution O 1s spectra. c) High-resolution Ti 2p spectra.


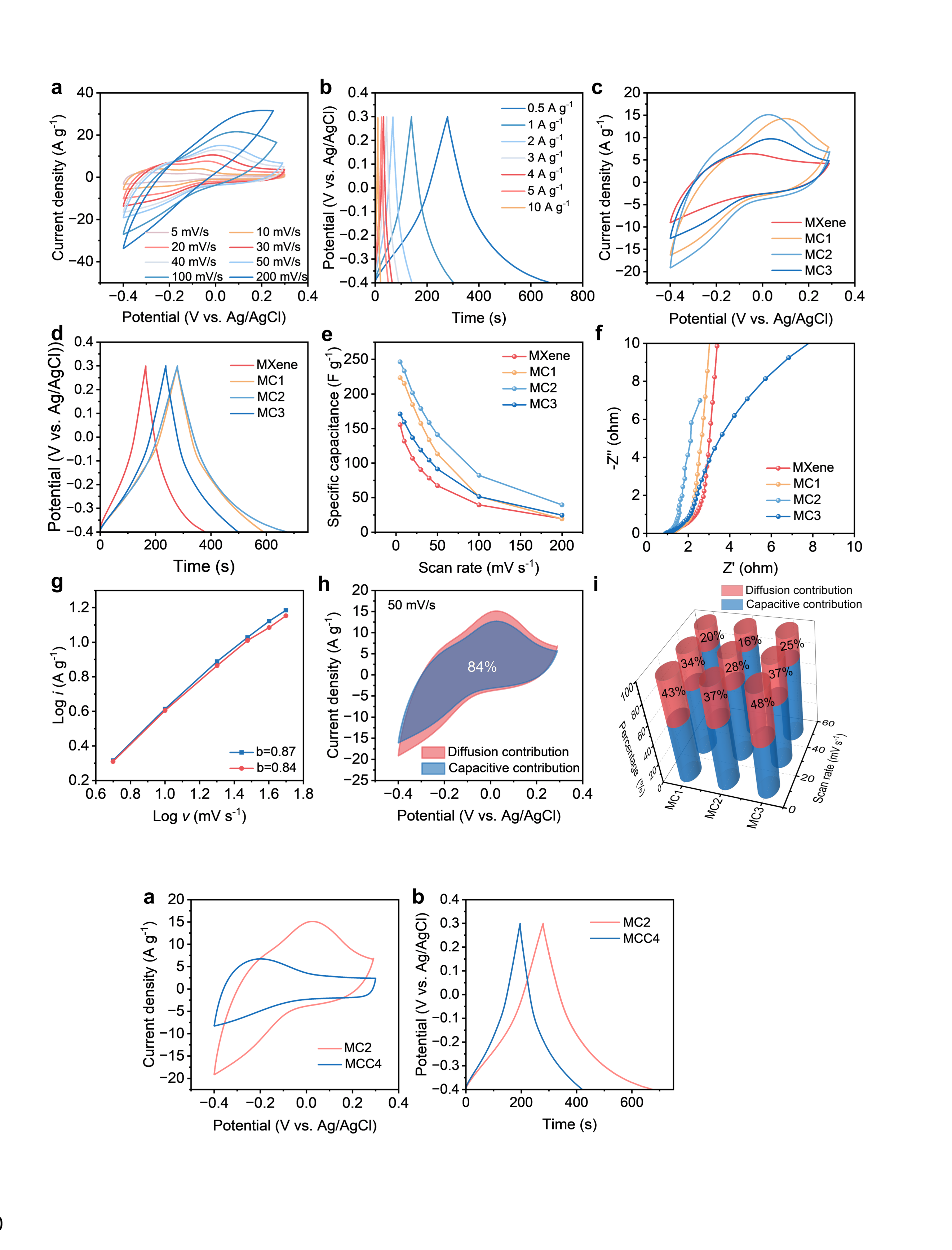


**Figure S11.** a) CV profiles 50 mV s^-1^ and b) GCD profile 0.5 A g^-1^ of MC2 and MCC4.


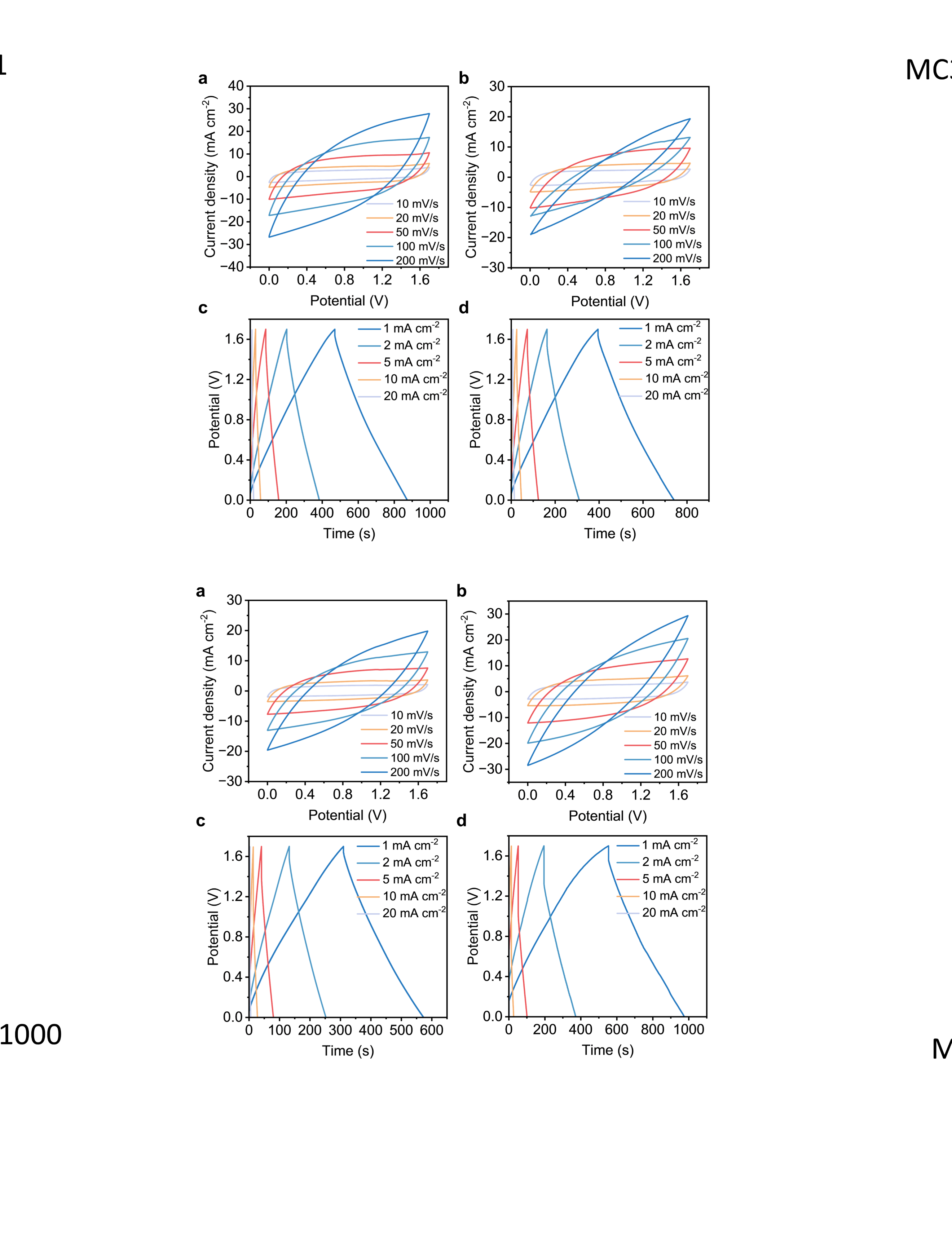


**Figure S12.** Electrochemical performances of all-solid-state supercapacitors based on MC electrodes with different ratios. a, b) CV profiles at various scan rates ranging from 10 to 200 mV/s of MC1 and MC3. c, d) GCD profile at various current densities ranging from 1 to 20 mA cm^-2^ of MC1 and MC3.


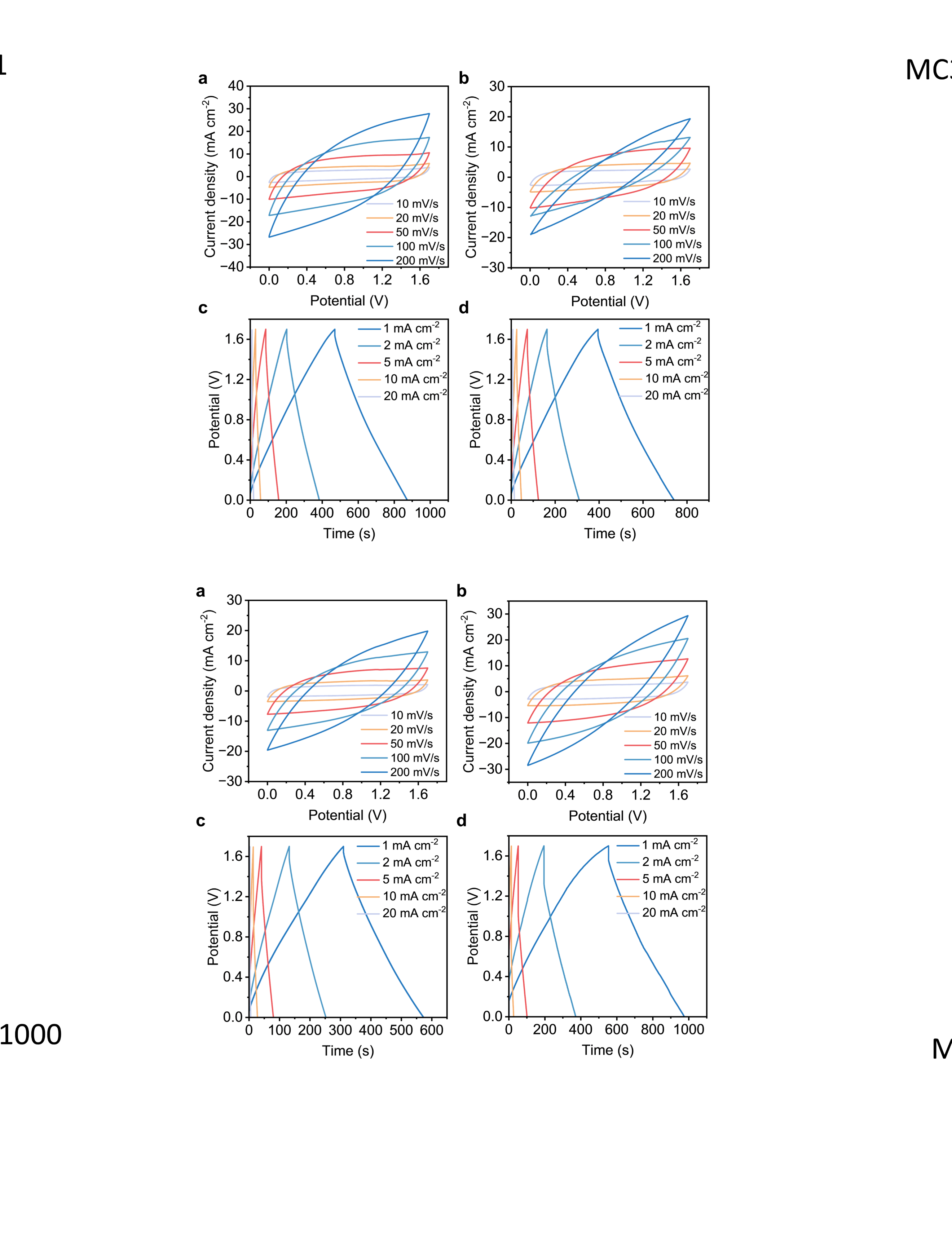


**Figure** **13.** Electrochemical performances of all-solid-state supercapacitors. a, b) CV profiles at various scan rates ranging from 10 to 200 mVs^-1^ of MXene/PH1000 and MXene/CNT. c, d) GCD profile at various current densities ranging from 1 to 20 mA cm^-2^ of MXene/PH1000 and MXene/CNT.


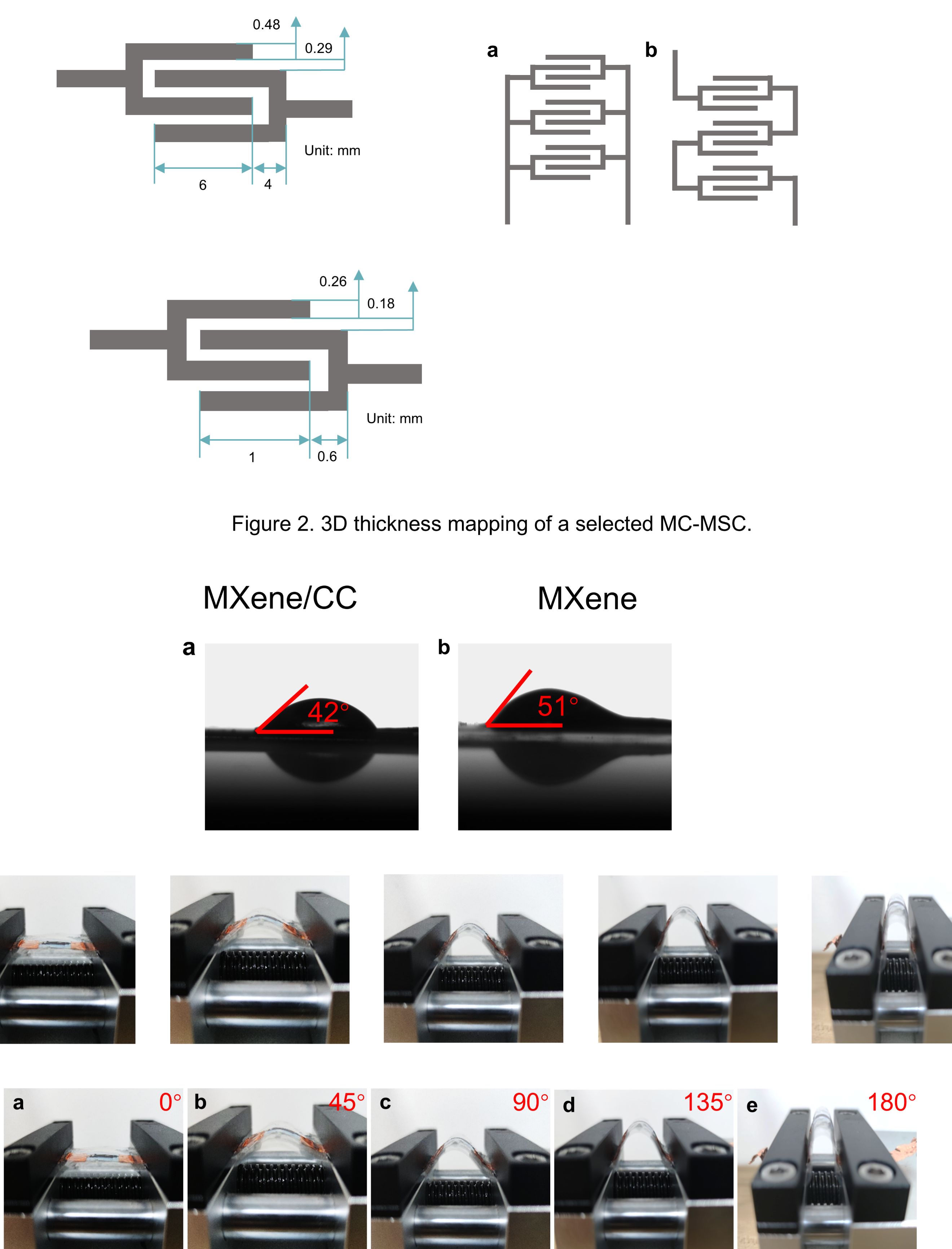


**Figure S14.** a) Contact angle between electrolyte and MC2 electrode. b) Contact angle between electrolyte and MXene electrode.


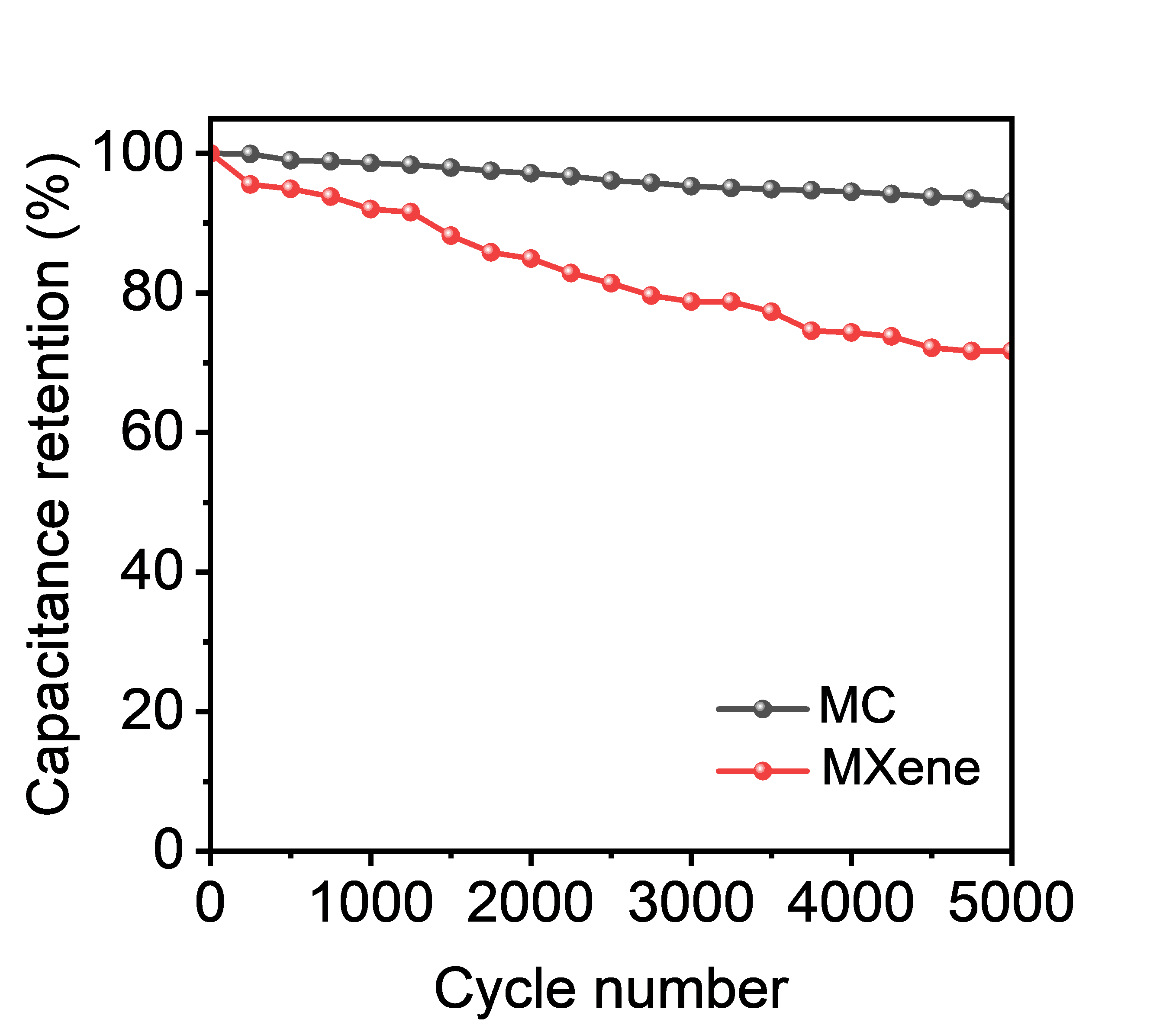


**Figure S15.** Cycling stability of MC and MXene electrode after long-term storage.


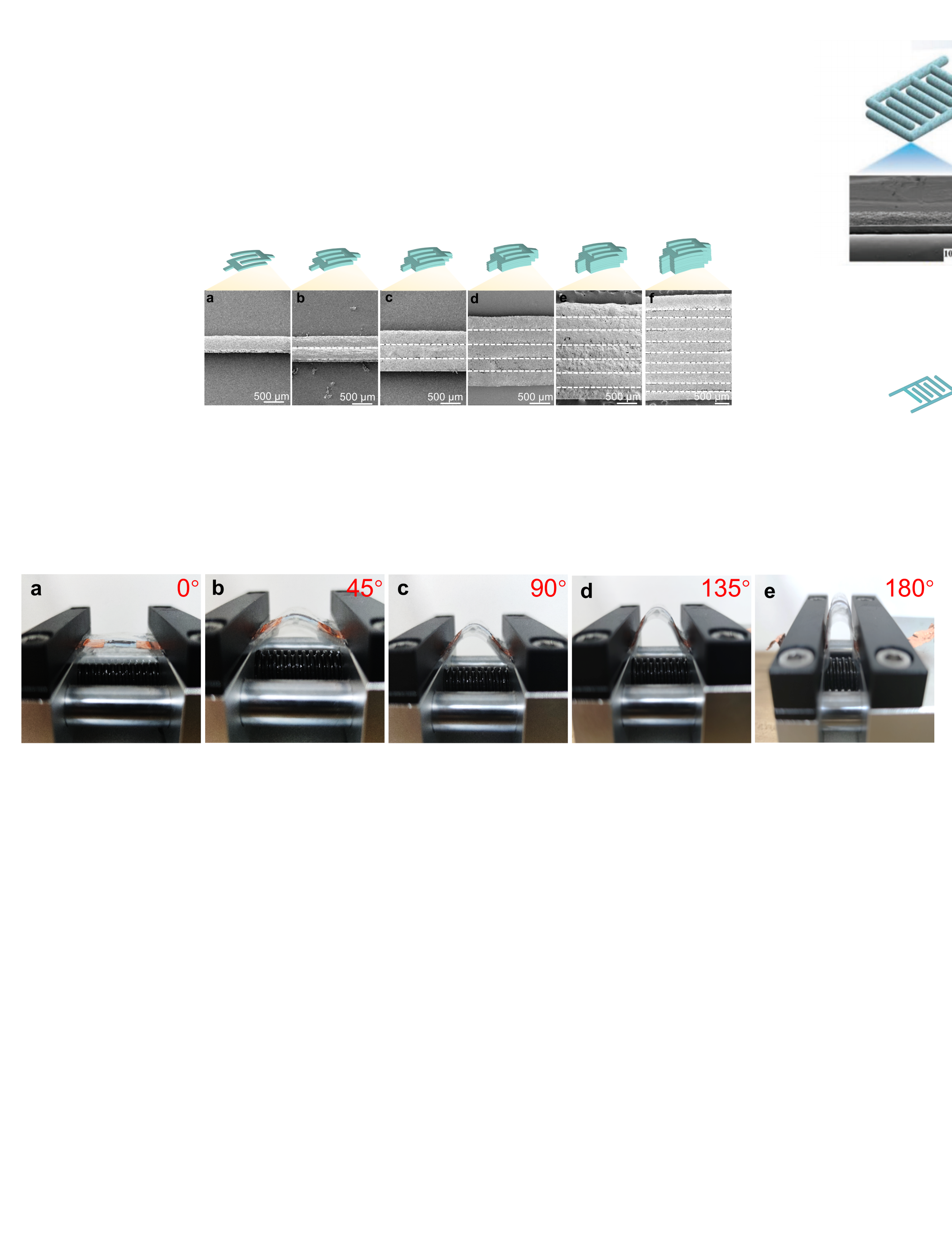


**Figure S16.** SEM images showing the thickness of the 3D printed MC2 electrode with different printing layers. a) 1 layer, b) 2 layers, c) 3 layers, d) 5 layers, e) 7 layers, and f) 10 layers.


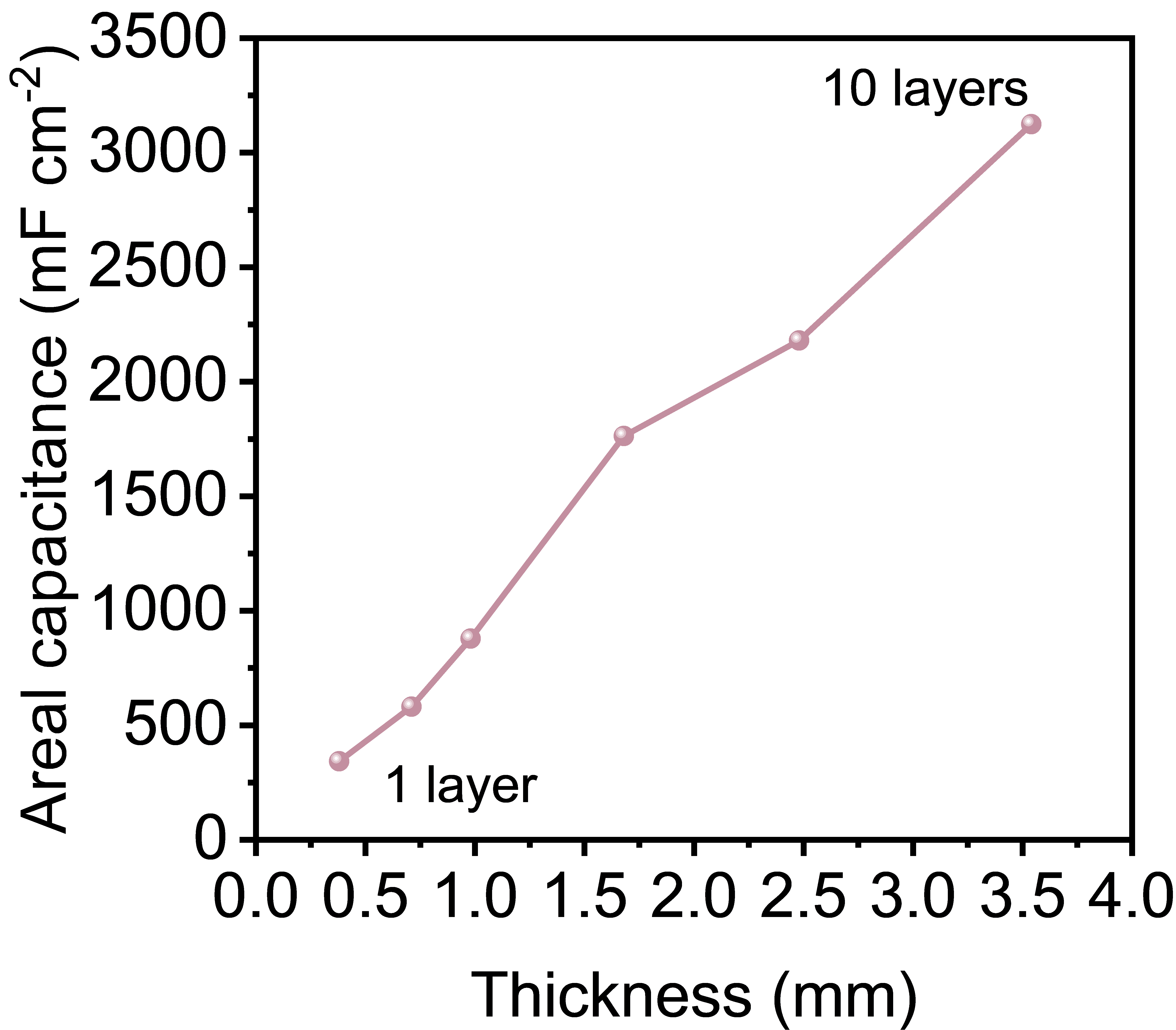


**Figure S17.** Areal capacitances of MC2-MSCs with various electrode thicknesses at a current density of 1 mA cm^-2^.


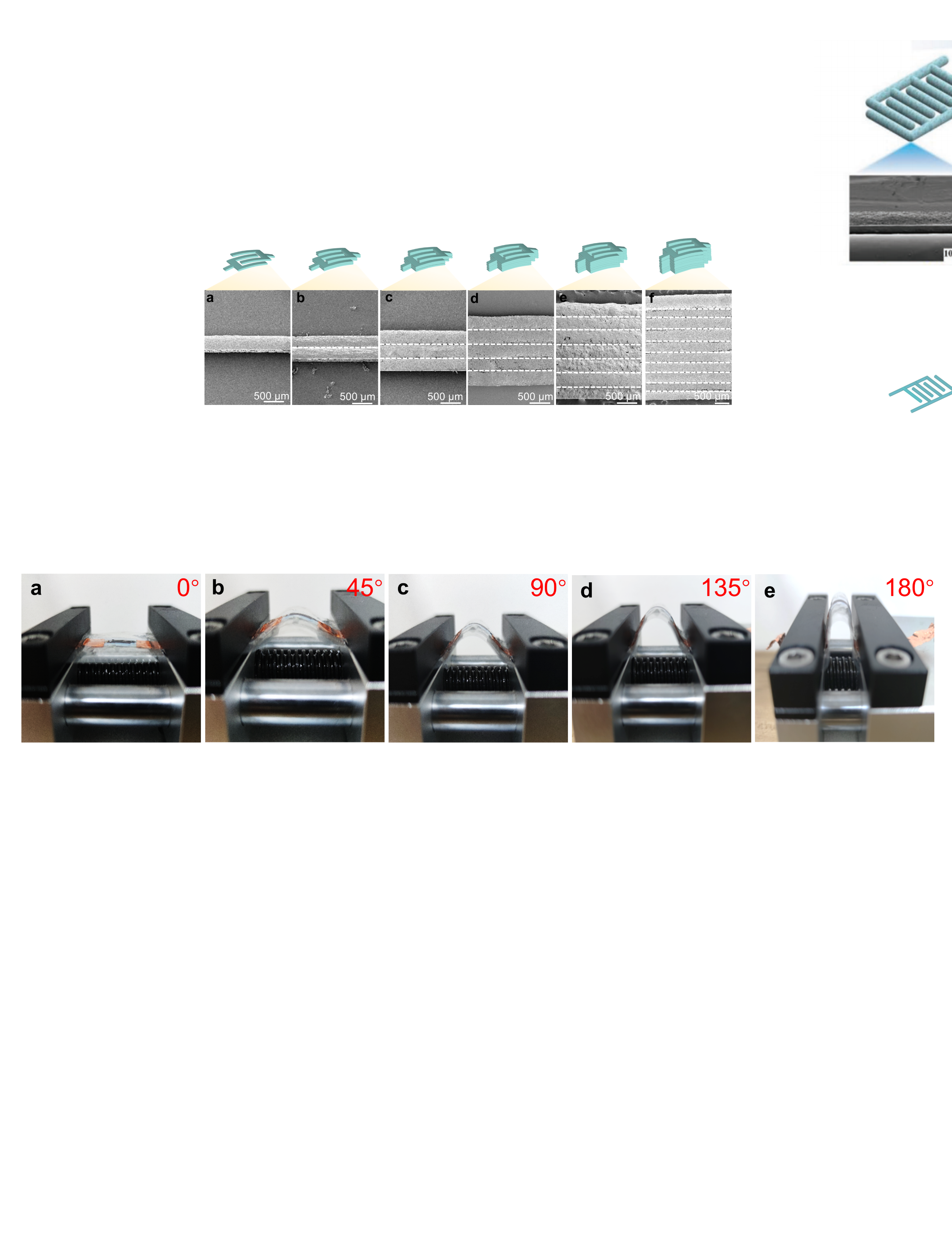


**Figure S18.** Optical images show that MC2-MSCs undergo bending deformation with different bending angles. a) 0°, b) 45°, c) 90°, d) 135°, and e) 180°.


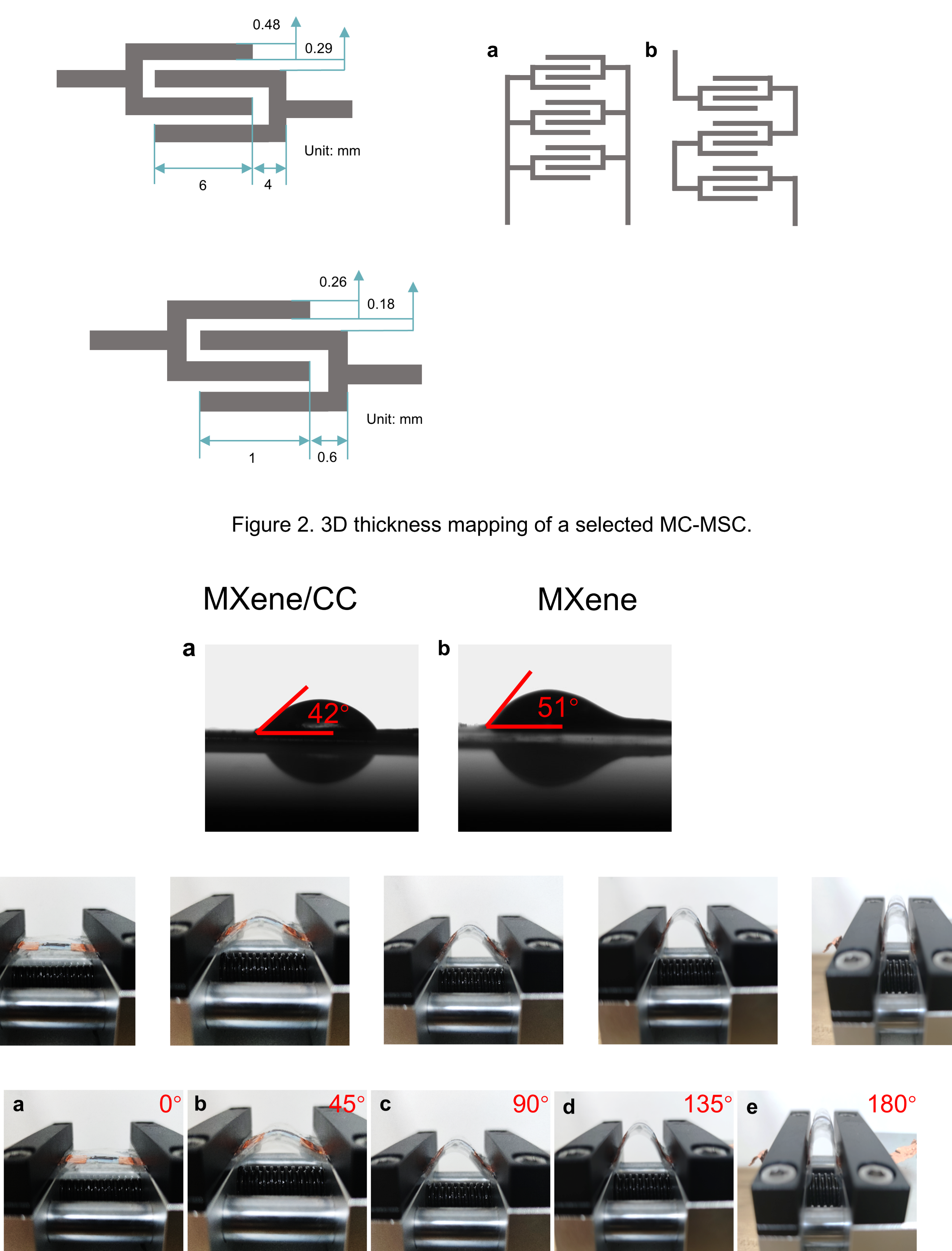


**Figure S19.** Schematic illustration of a) the microelectrode of 3D printed MC-MSC in parallel and b) series.


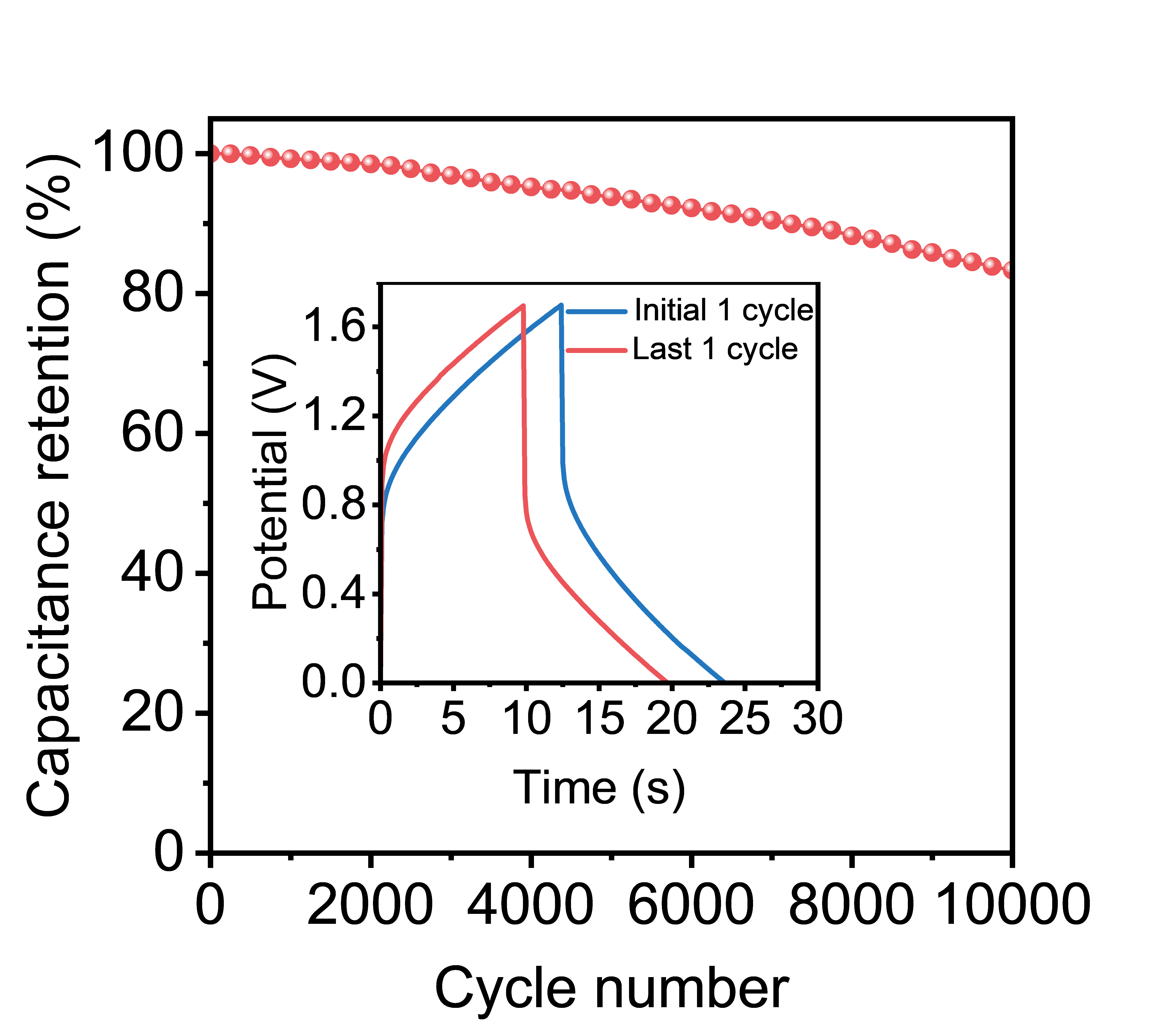


**Figure S20.** Cycling stability of MC under -20 °C (Inset: GCD profile at first cycle and after 10000 cycles).


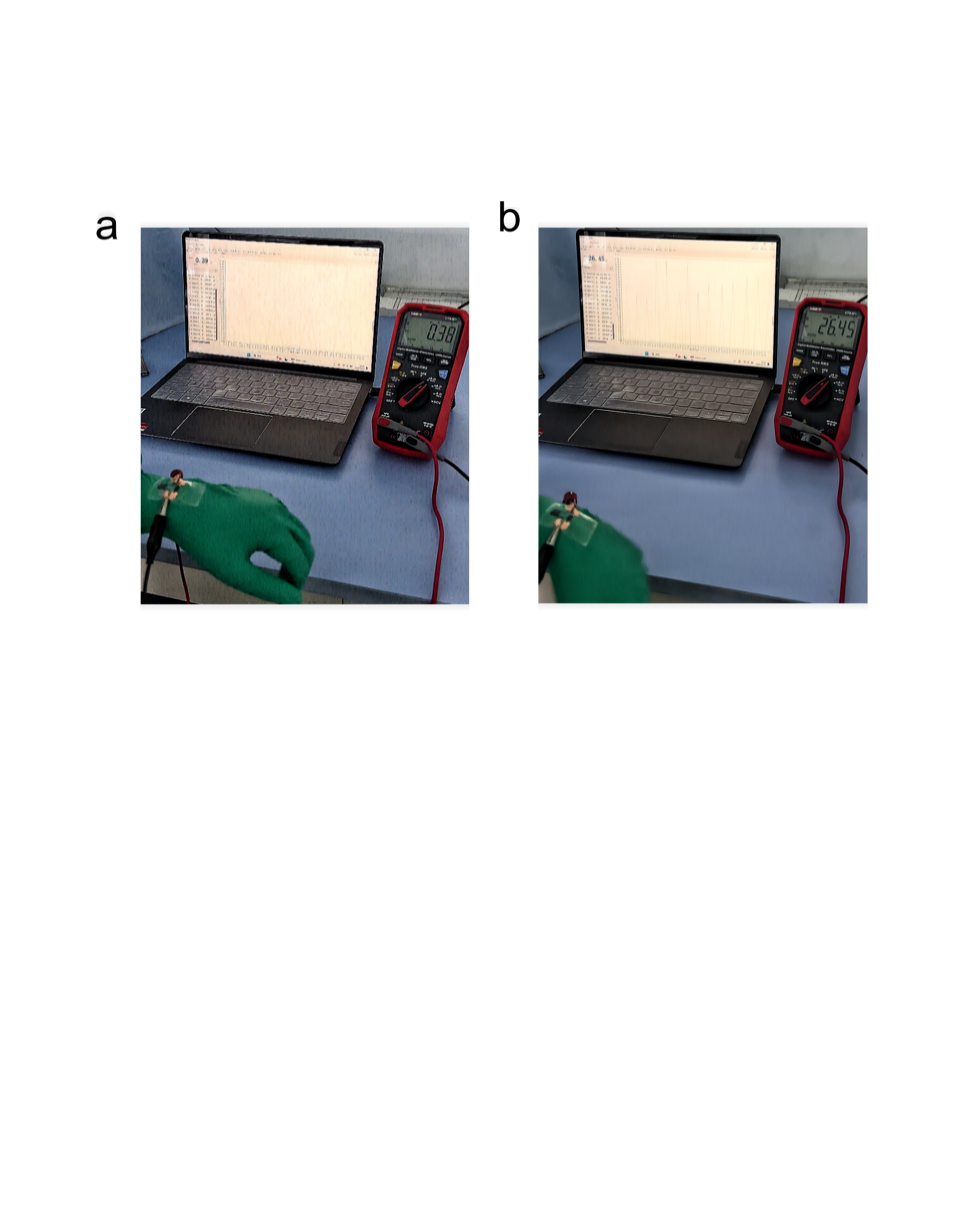


**Figure S21.** Photographs of MC2-MSC-sensor integrated system in the a) flat and b) bending states.

**Table S1.** Performance comparison of all-solid-state MSC devices with MXene-based electrodes

| **Electrode materials** | **Electrolyte** | **Voltage**  **(V)** | **Areal capacitance**  **(mF cm^-2^)** | **Energy density**  **(μWh cm^-2^)** | **Power**  **density**  **(mW cm^-2^)** | **Refs.** |
| --- | --- | --- | --- | --- | --- | --- |
| MC-MSC-1L | LiCl/SiO_2_ | 1.7 | 342.8  (1 mA cm^-2^) | 137.6 | 0.85 | **This work** |
| MC-MSC-10L | LiCl/SiO_2_ | 1.7 | 3124.6  (1 mA cm^-2^) | 1254.2 | 0.85 | **This work** |
| MXene/CNF  (12 L) | PVA/H_2_SO_4_ | 0.6 | 2020  (1 mA cm^-2^) | 101 | 0.299 | ^[1]^ |
| MXene sedimented | PVA/H_2_SO_4_ | 0.8 | 2337  (2 mV s^-1^) | 207.8 | 3.74 | ^[2]^ |
| MXene/PEDOT:PSS/EG  (10 L) | PVA/H_2_SO_4_ | 0.8 | 889  (2 mA cm^-2^) | 79 | 4 | ^[3]^ |
| MXene-BPEI  (10 L) | PVA/H_2_SO_4_ | 0.6 | 3783.53  (1 mV s^-1^) | 99.4 | 0.3 | ^[4]^ |
| MXene/CNF/MWCNT | PAM/CNF | 0.6 | 435  (1 mA cm^−2^) | 21.7 | 0.3 | ^[5]^ |
| Ti_3_C_2_T*_x_* MXene | PVA/H_2_SO_4_ | 0.6 | 2100  (1.7 mA cm^-2^) | 24.4 | 0.64 | ^[6]^ |
| MXene-AgNW-MnONW- | PVA/KOH | 0.8 | 216.2  (10 mV s^-1^) | 19.2 | 0.86 | ^[7]^ |
| Ti_3_C_2_T*_x_* MXene  (10 L) | PVA/H_2_SO_4_ | 0.6 | 1035  (2 mV s^-1^) | 51.7 | 5.7 | ^[8]^ |
| CNF/CNT/MXene | PVA/H_2_SO_4_ | 0.6 | 849.2  (0.8 mA cm^−2^) | 21.1 | 0.24 | ^[9]^ |

**References**

[1] G. Q. Zhou, M. C. Li, C. Z. Liu, Q. L. Wu, C. T. Mei, *Adv. Funct. Mater.* **2022**, *32*, 2109593.

[2] M. M. Yuan, L. B. Wang, X. Q. Liu, X. Y. Du, G. B. Zhang, Y. K. Chang, Q. X. Xia, Q. K. Hu, A. G. Zhou, *Chem. Eng. J.* **2023**, *451*, 138686.

[3] L. Li, J. Meng, X. R. Bao, Y. P. Huang, X. P. Yan, H. L. Qian, C. Zhang, T. X. Liu, *Adv. Energy Mater.* **2023**, *13*, 2203683.

[4] S. R. Liu, Q. Meng, Y. D. Gao, J. Z. Zhang, J. R. Li, Y. W. Yang, X. M. Zhang, H. P. Li, X. Y. Liu, *J.Mater. Chem. A* **2023**, *11*, 13238.

[5] G. Q. Zhou, X. Y. Liu, C. Z. Liu, Z. L. Li, C. H. Liu, X. J. Shi, Z. Y. Li, C. T. Mei, M. C. Li, *J. Mater. Chem. A* **2024**, *12*, 3734.

[6] W. J. Yang, J. Yang, J. J. Byun, F. P. Moissinac, J. Q. Xu, S. J. Haigh, M. Domingos, M. A. Bissett, R. A. W. Dryfe, S. Barg, *Adv. Mater.* **2019**, *31*, 1902725.

[7] X. R. Li, H. P. Li, X. Q. Fan, X. L. Shi, J. J. Liang, *Adv. Energy Mater.* **2020**, *10*, 1903794.

[8] J. Orangi, F. Hamade, V. A. Davis, M. Beidaghi, *ACS Nano* **2020**, *14*, 640.

[9] T. Xu, Q. Song, K. Liu, H. Y. Liu, J. J. Pan, W. Liu, L. Dai, M. Zhang, Y. X. Wang, C. L. Si, H. S. Du, K. Zhang, *Nano-Micro Lett.* **2023**, *15*, 98.
